# Gallic acid-grafted chitosan antibacterial hydrogel incorporated with polydopamine-modified hydroxyapatite for enhancing bone healing

**DOI:** 10.3389/fbioe.2023.1162202

**Published:** 2023-06-02

**Authors:** Yuxuan Pang, Lin Guan, Yanlin Zhu, Ruijuan Niu, Song Zhu, Quan Lin

**Affiliations:** ^1^ Department of Prosthodontics, School and Hospital of Stomatology, Jilin University, Changchun, China; ^2^ State Key Laboratory of Supramolecular Structure and Materials, College of Chemistry, Jilin University, Changchun, China; ^3^ Department of Oral Implantology, School and Hospital of Stomatology, Jilin University, Changchun, China; ^4^ Meilong Community Health Service Center, Shanghai, China

**Keywords:** hydrogel, polydopamine, gallic acid, antibacterial, bone regeneration

## Abstract

An open critical-size bone defect is a major medical problem because of the difficulty in self-healing, leading to an increased risk of bacterial infection owing to wound exposure, resulting in treatment failure. Herein, a composite hydrogel was synthesized by chitosan, gallic acid, and hyaluronic acid, termed “CGH.” Hydroxyapatite was modified with polydopamine (PDA@HAP) and introduced to CGH to obtain a mussel-inspired mineralized hydrogel (CGH/PDA@HAP). The CGH/PDA@HAP hydrogel exhibited excellent mechanical performances, including self-healing and injectable properties. Owing to its three-dimensional porous structure and polydopamine modifications, the cellular affinity of the hydrogel was enhanced. When adding PDA@HAP into CGH, Ca^2+^ and PO_4_^3-^ could release and then promoted differentiation of BMSCs into osteoblasts. Without any osteogenic agent or stem cells, the area of new bone at the site of defect was enhanced and the newly formed bone had a dense trabecular structure after implanting of the CGH/PDA@HAP hydrogel for 4 and 8 weeks. Moreover, the growth of *Staphylococcus aureus* and *Escherichia coli* was effectively inhibited through the grafting of gallic acid onto chitosan. Above, this study provides a reasonable alternative strategy to manage open bone defects.

## 1 Introduction

Large bone defects caused by trauma seriously affect the physical and psychological health of patients (Xie et al., 2020). Although autologous grafts and allografts are widely used as the gold-standard bone repair materials for clinical treatment, they are associated with numerous disadvantages, including immune rejection, surgical trauma, and limited biological activity (Wang et al., 2022). Use of a scaffold exhibiting desirable biomechanical properties, degradability, and osteogenic activity is considered a reasonable method to promote bone regeneration in the field of tissue engineering. Hydrogels have been developed with these advantageous properties, which have been shown to effectively accelerate the bone healing process, thus offering great application value (Sahiner et al., 2016; Guan et al., 2019; Liu et al., 2022). In particular, the extracellular matrix-like structure and properties of hydrogels conferred by loading osteogenically active substances can effectively regulate the adhesion, diffusion, and osteogenic differentiation of bone marrow-derived mesenchymal stem cells (BMSCs), thus laying a solid foundation for the subsequent formation of bone tissue (Sun et al., 2016; Liu et al., 2018).

Hydroxyapatite (HAP) is mainly responsible for the support, protection, and carrying capacity of natural bone tissue, and organic components such as type I collagen, type III collagen, and fibrin mainly control the proliferation, migration, and differentiation of BMSCs (Gao et al., 2018; Leng et al., 2020). Accordingly, biomineralized hydrogels with HAP as the main active ingredient have been manufactured that exhibit desirable hydrophilicity, degradability, and mechanical strength (Zhao et al., 2020; Ren et al., 2022; Wan et al., 2022). Such HAP-based hydrogels not only positively regulate the biological behavior of BMSCs but also provide a suitable biochemical environment for the osteogenic differentiation of BMSCs via the continuous release of Ca^2+^ and PO_4_^3-^ from HAP (Lee et al., 2014; Chen et al., 2016; Gao et al., 2022). However, HAP is prone to aggregation in hydrogels, leading to high concentrations during degradation, which interfere with the adhesion, proliferation and osteogenic differentiation of BMSCs (Jiang et al., 2017; Pan et al., 2020; Xu et al., 2020). Thus, improving the biocompatibility of HAP-based hydrogels is an important clinical need.

Inspired by the proteins responsible for the strong adhesion property of mussels, polydopamine (PDA) has been applied in the functionalization of biomaterials to bind with Ca^2+^ owing to its catechol structure (Zhou et al., 2012; Peng et al., 2014). Through PDA coating, HAP is evenly distributed on materials, which can avoid its aggregation and excessive ion concentration (Gao et al., 2022). Furthermore, the catechol group of PDA has strong surface activity for nucleophilic functional groups, which can promote cell growth onto the materials (Cui et al., 2012; Park et al., 2014; Wang et al., 2021a). This effect can be attributed to the ability of PDA to increase the overall positive charge and thus improve the hydrophilicity of the material (Zhang et al., 2022b; Li et al., 2022).

Post-traumatic bone infection is one of the common complications of an open bone defect because an open wound provides the opportunity for *Staphylococcus aureus* and other Gram-negative pathogenic bacteria to invade the defect area, leading to bone destruction and non-union (Lüthje et al., 2020). Although surgical debridement and postoperative application of antibiotics reduce the probability of infection to some extent, the disadvantages of a longer treatment cycle and generation of drug-resistant strains cannot be ignored, and bacteria colonizing deep areas of the wound are often difficult to detect (Wang et al., 2021b). Therefore, introducing an active substance with inherent antibacterial properties into scaffolds is a reasonable modification strategy (Han et al., 2019; Chen et al., 2021; Yang et al., 2021). Gallic acid (GA) has been widely applied for antibacterial modification of biomaterials given its ability to destroy the integrity of the bacterial cell wall and interfere with the activity of the respiratory electron transport chain (Shi et al., 2021). Moreover, catechol groups on the surface of GA react actively with various functional groups to promote cell adhesion, providing multiple possibilities for its application in hydrogel grafting (Asha et al., 2022).

**SCHEME 1:**
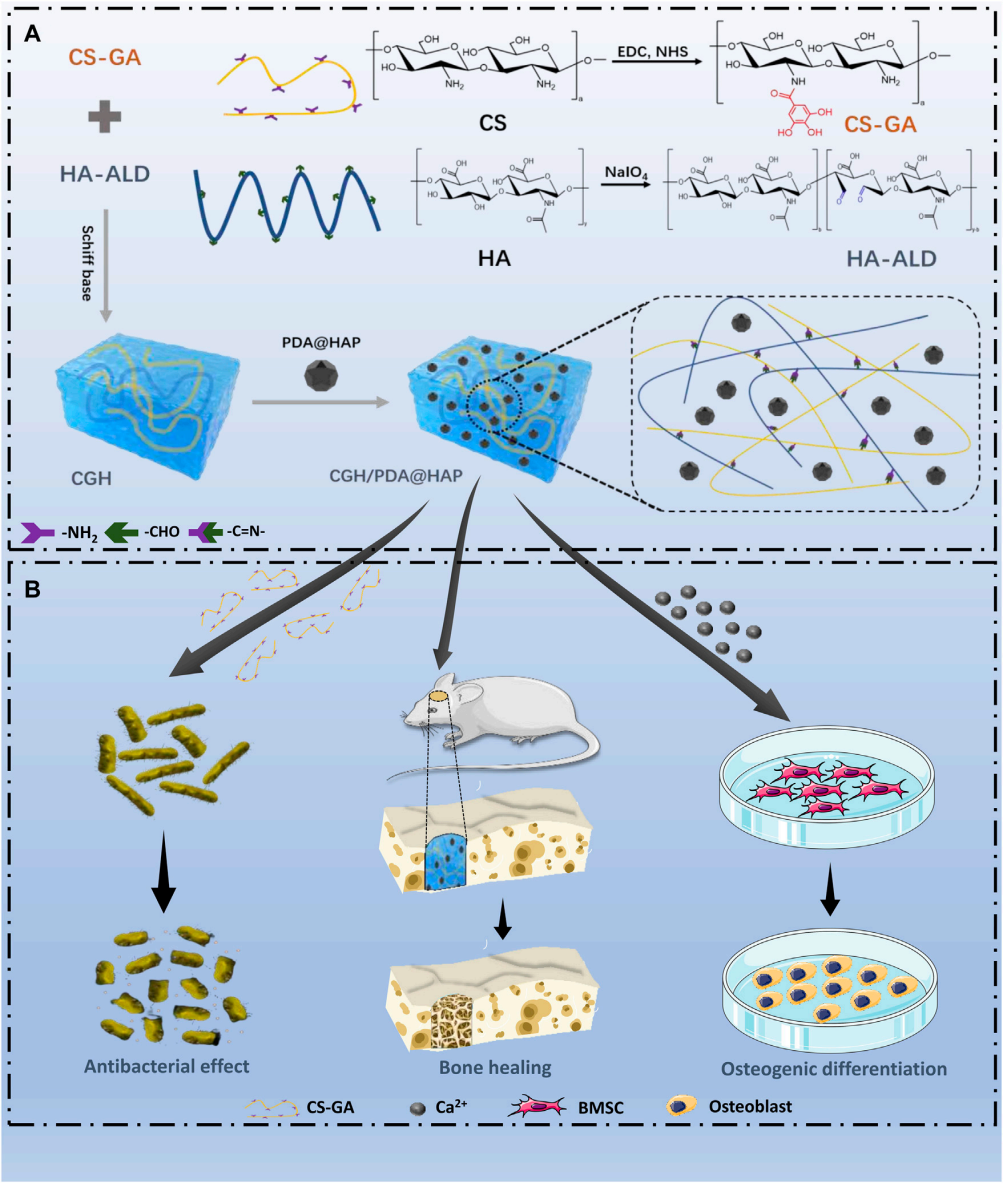
Preparation and biological effects of the CGH/PDA@HAP hydrogel. **(A)** Synthetic process **(B)** Biological effects.

In this study, we developed a mussel-inspired mineralized hydrogel based on GA-grafted chitosan (CS) by introducing PDA-modified HAP (PDA@HAP), which exhibited excellent osteogenic and antibacterial properties (Scheme 1). Specifically, this hybrid hydrogel (CGH/PDA@HAP) was fabricated from the assembly of CS-grafted GA (CG), hyaluronic acid (HA), and PDA@HAP. The three-dimensional porous structure of the CGH/PDA@HAP hydrogel allows BMSCs to adhere to and proliferate on its surface, showing good biocompatibility. The HAP is evenly distributed along the hydrogel owing to the grafted PDA, thereby avoiding aggregation and stabilizing the ion concentration in the local environment. As a result, PDA@HAP effectively promoted the differentiation of BMSCs into osteoblasts and upregulated the expression of osteoblast-related genes *in vitro*. Further, imaging with micro-computed tomography (micro-CT) and histological observations with hematoxylin-eosin (HE) and Masson staining revealed the desirable therapeutic effect of the CGH/PDA@HAP hydrogel on rat calvarium defects. Finally, GA grafting clearly improved the antibacterial properties of the hydrogel, preventing bacterial infection during bone regeneration *in vivo*.

## 2 Materials and methods

### 2.1 Preparation and characterization of the CGH/PDA@HAP hydrogel

#### 2.1.1 Preparation of PDA@HAP

For PDA functionalization of HAP, 90 mL of Tris-hydrochloride buffer (pH 8.5; Aladdin, China) was mixed with 10 mL of ethanol. Subsequently, 2 g of HAP (Aladdin, China) was dispersed over the solution by ultrasound. Dopamine hydrochloride (1 mg/mL; Aladdin, China) was then added to the above solution and stirred in the dark for 24 h. The unreacted dopamine hydrochloride was removed by washing with water and centrifugation, and the product was ultrasonically dispersed. Finally, PDA@HAP was obtained by freeze-drying.

#### 2.1.2 Preparation of oxidized HA (HA-ALD)

One Gram of HA (Yuanye, China) was uniformly dissolved in 100 mL of H_2_O, followed by the addition of 1 g of sodium periodate (NaIO_4_; Sigma, America), which was left to react for 3 h in the dark. Subsequently, 3 mL ethylene glycol (Beijing Chemical Works, China) was added to the solution and stirred for 1 h to terminate the reaction. The obtained solution was dialyzed in deionized water for 3 days and the water was changed at least three times a day. Finally, the solution was freeze-dried to obtain HA-ALD.

#### 2.1.3 Preparation of GA-modified CS (CS-GA)

CS (Aladdin, China) was dissolved in water and adjusted to form a 1 wt% solution with 5 M HCl. The solution was heated in a water bath and deoxidized with N_2_ for 15 min. GA (Aladdin, China) was then dissolved in the CS solution. The N-(3-Dimethylaminopropyl)-N′-ethylcarbodiimide hydrochloride (EDC; Aladdin, China) and N-Hydroxysuccinimide (NHS; Aladdin, China) dissolved in 50 mL H_2_O were dropped into the GA and CS mixture, and left to react for 10 h in the N_2_ environment while maintaining the pH at 5. The obtained solution was dialyzed with acidified deionized water for 3 days to inhibit oxidation of the catechol group. Finally, the CS-GA powder was obtained by freeze-drying.

#### 2.1.4 Preparation of the CGH/PDA@HAP hydrogel

The 1 wt% HA-ALD solution was dissolved in a 2 wt% CS-GA solution, and then 1 wt% PDA@HAP was dissolved in a 5 wt% HA-ALD solution and uniformly distributed under ultrasonic stirring. The two solutions were then mixed and shaken to obtain a uniform GGH/PDA@HAP hydrogel. CS hydrogels without the GA modification were prepared under the same process for comparison.

#### 2.1.5 Characterization of the CGH/PDA@HAP hydrogel

The synthesized hydrogel was characterized by Fourier-transform infrared (FTIR) spectra obtained on a BLUCK spectrophotometer over a wavenumber range from 4000 to 500 cm^-1^, ultraviolet (UV)-visible absorption spectra measured with a Lambad 800 spectrophotometer, and H-nuclear magnetic resonance (NMR) spectroscopy on a BLUCK AVANCEIII600 device. The dynamic mechanical properties of the hydrogel were determined with a rheometer (TA Instruments-waters LLC). The morphology of the hydrogels was observed by cryo-scanning electron microscopy (Cryo-SEM; Zeiss Sigma-300, Germany) at −140°C. X-ray diffraction (XRD; Rigaku, Japan) was used to analyze the phase compositions of the CGH and CGH/PDA@HAP hydrogels. The swelling ratio of the hydrogels was measured in PBS at 37°C. For enzymatic degradation assay, the CGH and CGH/PDA@HAP hydrogels were incubated in the PBS solution containing hyaluronidase (900U/mL; Solarbio, China) at 37°C for 21 days. The CGH/PDA@HAP hydrogel was immersed in sterile deionized water for 5 days and inductively coupled plasma emission spectrometer (ICAP-7400; ThermoFisher, America) was used to an investigate the calcium (Ca^2+^) and phosphorus (PO_4_^3-^) release.

### 2.2 Cytotoxicity and cell proliferation assay

#### 2.2.1 Cell culture

The BMSCs were isolated from three-week-old male Sprague-Dawley (SD) rats (Changsheng Biotechnology, China) and cultured in low-glucose Dulbecco’s modified Eagle medium (LG-DMEM; Solarbio, China) supplemented with 10% fetal bovine serum (FBS; Genemini, United States) and 1% penicillin-streptomycin (P-S; Hyclone, United States) at 37°C in a 5% CO_2_ atmosphere. BMSCs were used at passage 3 for further *in vitro* experiments.

To assess the effects of the hydrogel on BMSCs, the culture medium was supplemented with CGH and CGH/PDA@HAP. Briefly, the hydrogels were immersed in LG-DMEM and cultured for 48 h (Xu et al., 2020). The extracts were filtered for sterilization and the cells were subsequently cultured in osteogenic medium (85% LG-DMEM, 10% FBS, 1% P-S, 50 μM ascorbate, 100 mM β-sodium glycerophosphate, 100 nM dexamethasone).

#### 2.2.2 Cell live/dead staining

The Calcein AM/propidium iodide (PI) cell live and dead staining kit (Yeasen, China) was used to observe the survival of BMSCs for evaluating the cytotoxicity of CGH and CGH/PDA@HAP hydrogels. In brief, the bottom of a 6-well plate was covered with the prepared hydrogel, sterilized by UV light, cleaned in deionized water, and pre-soaked in DMEM before the experiment. The hydrogel was then inoculated with 5 × 10^4^ BMSCs. After culturing for 24 h, the fluorescence staining solution (2 μM Calcein AM and 4.5 μM PI) was added, incubated for 15 min, and imaged by a live cell imager.

#### 2.2.3 Proliferation assay

To further assess the effect of the hydrogels on the proliferation of BMSCs, the Cell Counting Kit-8 (CCK-8; Yeasen, China) assay was used to evaluate the number of live BMSCs in the hydrogels. The hydrogel was pre-treated as described above. Subsequently, 5 × 10^3^ BMSCs were seeded onto the surfaces of the hydrogels in a 96-well plate and the complete medium was replaced every 3 days. The cell proliferation was assessed at 1, 4, and 7 days. At each time point, the medium was replaced with fresh basic medium and CCK-8 reacting solutions were added. After incubation for 2 h, the optical density (OD) value was measured at 450 nm with a microplate reader. BMSCs cultured only in complete medium were used as the control group.

### 2.3 Osteogenic differentiation assay *in vitro*

#### 2.3.1 Alkaline phosphatase (ALP) staining

ALP is a marker of early osteogenesis that is often used to evaluate the degree of the early osteogenic differentiation of BMSCs (Wang et al., 2021a). ALP staining and corresponding quantitative analysis were performed to evaluate the effect of the hydrogel on the osteogenic differentiation ability of BMSCs in the early stage. Briefly, BMSCs were cultured in the extract-modified osteogenic medium for 14 days, washed twice by phosphate-buffered saline (PBS), and then fixed with 4% paraformaldehyde for 30 min. The staining procedure was performed according to the protocol described in the kit instructions (Beyotime, China). After staining, BMSCs were washed with PBS and observed under a light microscope, and images were captured. A quantification kit (Jiancheng, China) was also used to quantify the ALP activity in the BMSCs. Briefly, BMSCs were fully lysed and the protein supernatant was obtained to measure the ALP activity. A bicinchoninic acid (BCA) kit (Beyotime, China) was used to normalize the total protein concentration. BMSCs incubated in osteogenic medium alone were set as the control group.

#### 2.3.2 Alizarin Red staining (ARS)

ARS was used to observe the mineralization in the extracellular matrix of BMSCs. Briefly, after culture for 21 days, BMSCs were washed with PBS, fixed with 4% paraformaldehyde for 30 min, and then ARS reaction solution (Solarbio, China) was added to the 6-well plate. After incubating in the dark for 30 min, the solution was aspirated and the 6-well plate was washed with deionized water three times, followed by image capture and analysis. Quantification of mineralization in the extracellular matrix was performed using 10% cetylpyridinium chloride (Aladdin, China) to dissolve the cells stained by Alizarin Red. The OD value was measured at 562 nm using a microplate reader. BMSCs incubated in osteogenic medium alone were set as the control group.

#### 2.3.3 Reverse transcription-quantitative polymerase chain reaction (RT-qPCR)

RT-qPCR was used to evaluate the osteogenic differentiation of BMSCs according to the mRNA expression of the osteogenic markers Runt-related transcription factor-2 (*Runx2*), alkaline phosphatase (*Alp*), and osteocalcin (*Ocn*). Briefly, after culture for 7 and 14 days, BMSCs were washed with PBS and total RNA was isolated by Trizol Reagent (Yeasen, China) according to the manufacturer instructions. The concentration and purity of RNA were measured by a NanoDrop 2000c spectrophotometer (ThermoFisher Scientific, United States). RNA with a concentration of 100–1000 ng/μL and an absorbance ratio at 260/280 nm of approximately 1.8–2.2 was considered to be of suitable quality for further analysis. The total RNA was reverse-transcribed into cDNA using Strand cDNA Synthesis SuperMix kit (Yeasen, China). The cDNA was then used as the template for qPCR with SYBR Green Master Mix (Yeasen, China) and primers for the target gene, and the results were analyzed on a Lightcycler 960 system. The primers were synthesized by Sangon Biotech and their sequences are shown in Table 1; β-actin served as housekeeping gene for normalization. The 2^**–△△**Ct^ method was used to compare relative mRNA expression levels between experimental and control groups. BMSCs incubated in osteogenic medium alone were set as the control group.

**TABLE 1:** Primer sequences for RT-qPCR.

Primer	Forward sequence (5′–3′)	Reverse sequence (5′–3′)
*Runx2*	CTTCGTCAGCGTCCTATCAGTTCC	TCCATCAGCGTCAACACCATCATTC
*Alp*	GGC​GTC​CAT​GAG​CAG​AAC​TAC​ATC	CAG​GCA​CAG​TGG​TCA​AGG​TTG​G
*Ocn*	GGA​CCC​TCT​CTC​TGC​TCA​CTC​TG	ACC​TTA​CTG​CCC​TCC​TGC​TTG​G
β-actin	TGTCACCAACTGGGACGATA	GGGGTGTTGAAGGTCTCAAA

### 2.4 Bone regeneration assay *in vivo*

#### 2.4.1 Rat calvarium defect model establishment and hydrogel implantation

All animal studies were approved by the Institutional Animal Care and Use Committee of Jilin University (IACUC No. SY202203100). A critical-sized rat calvarium defect was made to evaluate the osteogenesis effects of the CGH/PDA@HAP hydrogel *in vivo*. Eighteen male SD rats with a weight of 220–250 g were weighed and anesthetized by intraperitoneal injection of pentobarbital sodium (45 mg/kg). After successful anesthesia, the rats were fixed in prone position. After routine preparation of the cranial skin, 1% iodophor and 75% alcohol were used for disinfection and sterile towels were spread. A 1.5-cm incision was made along the sagittal line of the skull. The skin and periosteum were dissected, and the periosteum was separated with an exfoliator to fully expose the skull. An annular bone drill was connected to the implant base, and a bone defect of 5 × 5 mm was prepared by drilling a hole (drilling rate <1500 rpm) in the outer lower quadrant of the skull where the median suture and the herringbone suture crossed. During the operation, normal saline was used to avoid overheating that could cause local tissue necrosis and to avoid the damage of brain tissue due to drilling too deep. After confirming successful model establishment, the hydrogels were injected at the same time, and the periosteum, subcutaneous tissue, and epidermis were sutured in sequential layers. The rats were placed in a warm and ventilated cage until they regained consciousness and crawled on their own. After the operation, aspirin (100 mg/kg) was dissolved in the drinking water for free ingestion of analgesia for no less than 3 days. The rats were divided into three groups: CGH, CGH/PDA@HAP, and control group (without any hydrogel).

#### 2.4.2 Micro-CT analysis

To detect the new bone regenerated at defect sites, micro-CT (SCANCO, Switzerland) was employed for observation and quantitative analysis. Briefly, three-dimensional reconstruction of samples of each group was performed, and the bone volume/tissue volume (BV/TV) and trabecular separation (Tb.Sp) were automatically determined according to manufacturer’s operational protocol for further evaluating the healing effect on bone defects of each group.

#### 2.4.3 Histological evaluation

All samples were decalcified with 15% ethylenediaminetetraacetic acid (pH 7.2) for 4 weeks. The dehydrated specimens were embedded in paraffin and cut into 5-μm continuous sections for staining. The pathological sections of each group were evaluated by HE and Masson’s trichrome staining, and the films were taken under a light microscope. Additionally, the major organs, including the heart, liver, spleen, lung, and kidney, were harvested for HE staining to evaluate the biosafety of the hydrogels.

### 2.5 Antibacterial assay

#### 2.5.1 Bacterial strains and culture

*Staphylococcus aureus* (ATCC29213; Fenghui, China) and *Escherichia coli* (ATCC25922; Fenghui, China) were selected in this study. Briefly, each species was incubated with Luria-Bertani (LB) medium at 37°C under relative humidity above 90%. Based on the OD value measured at 600 nm in a UV spectrophotometer, the bacterial suspension was adjusted to 1 × 10^6^ colony-forming units (CFU)/mL for subsequent experiments.

#### 2.5.2 Planktonic bacteria inhibition assay

Time-kill curves construction and bacterial colony counting were applied to evaluate the bacterial inhibitory ability of the CGH/PDA@HAP and CGH hydrogels. Briefly, hydrogels (200 μL) were co-cultured with the bacterial suspension (500 μL) in 24-well plates for 12 h. The OD value was measured at 600 nm every 2 h for 12 h and time-kill curves were constructed. Subsequently, the suspension treated with the hydrogel materials was diluted to a certain ratio, and a 30 μL solution was evenly spread onto agar LB plates. The colonies were observed and the CFU was calculated after culturing for 48 h. *Staphylococcus aureus* and *E. coli* simply cultured in LB medium were set as the control groups.

#### 2.5.3 Bacteria live/dead staining assay

The SYTO 9/PI (live/dead) staining kit (ThermoFisher, America) was used to determine the survival status of *S. aureus* and *E. coli* cultured on CGH/PDA@HAP and CGH hydrogels. Briefly, the bacteria were cultured as described above. SYTO 9 (2.5 mM) and PI (2.5 mM) were mixed to form a reacting solution, added onto the surface of the hydrogel, and then left to react in the dark for 15 min. Images were observed and captured with an inverted fluorescence microscope.

#### 2.5.4 Metabolic activity

The 3-(4,5-dimethylthiazol-2-yl)-2,5-diphenyltetrazolium bromide (MTT; Beyotime, China) assay was used to evaluate the biofilm activity of *S. aureus* and *E. coli* when co-cultured with the CGH and CGH/PDA@HAP hydrogels. Briefly, 0.4 mL MTT solution (0.5 mg/mL) (Beyotime, China) was added onto the hydrogel in a 24-well plate and incubated for 2 h at 37°C. Equal amounts of dimethyl sulfoxide was added to each well to dissolve the formaldehyde crystals after reacting in the dark. The MTT dye was transferred onto a new 96-well plate and the OD value was measured at 540 nm in a standard enzyme assay instrument.

### 2.6 Statistical analysis

Quantitative values are presented as mean ± standard deviation from at least three independent experiments and differences among groups were analyzed using one-way analysis of variance (GraphPad Prism 8.0.1 software) to assess statistical significance. *p* < 0.05 was considered statistically significant.

## 3 Results and discussion

### 3.1 Preparation of the CGH/PDA@HAP hydrogel

**FIGURE 1:**
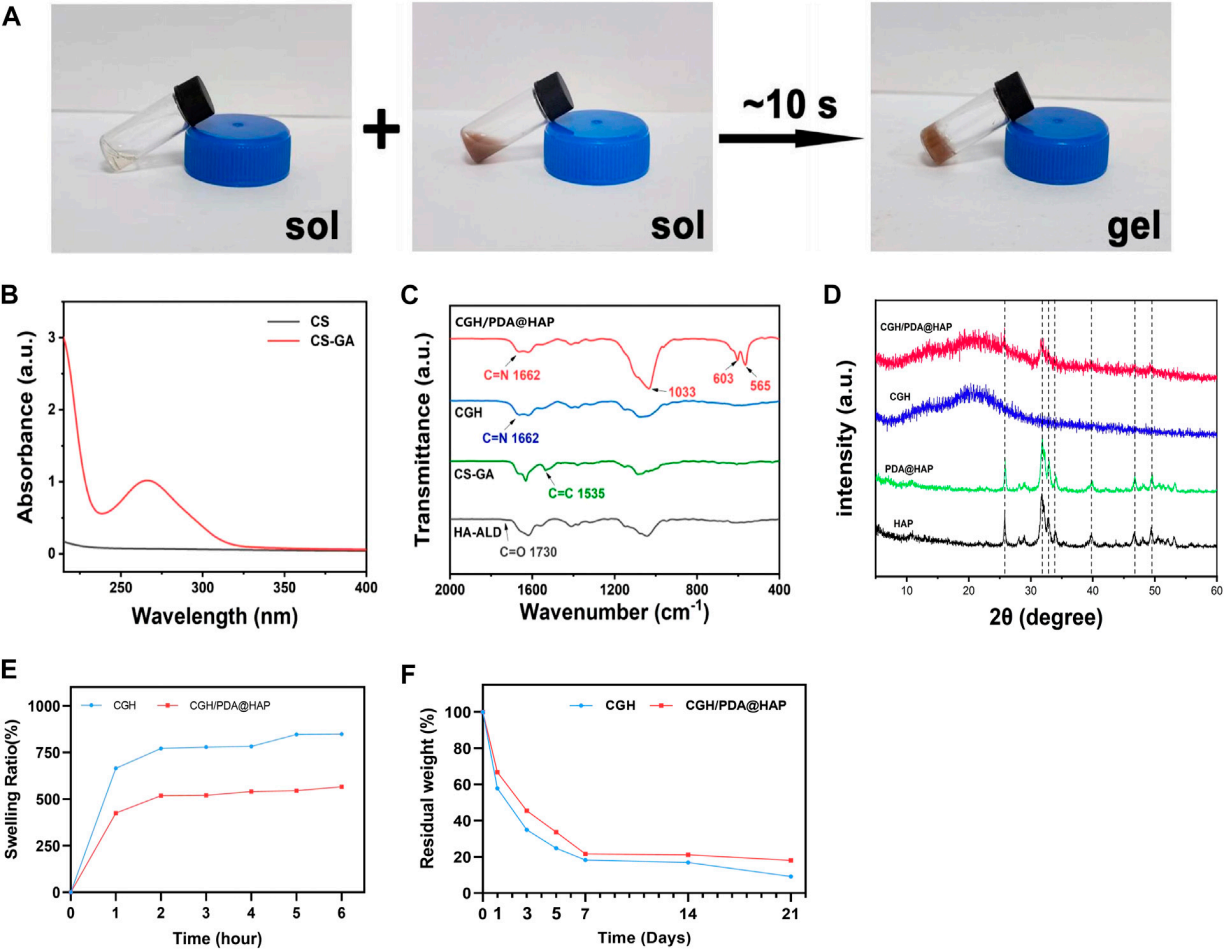
Preparation of the CGH/PDA@HAP hydrogel. **(A)** Optical image of the gelation process. **(B)** UV absorbance spectrum of CS-GA. **(C)** FTIR spectra. **(D)** XRD analysis. **(E)** Swelling ratio. **(F)** Enzymatic degradation.

To overcome the problem of poor HAP dispersion, we modified the surface of HAP with PDA (PDA@HAP) to effectively improve its hydrophilicity, which is mainly achieved due to the large number of hydrophilic groups on the surface of PDA (Wu et al., 2019; Gao et al., 2022). The successful preparation of the PDA@HAP material was confirmed by the FTIR spectra (Supplementary Figure S1). Moreover, we synthesized CS-GA by the amidation reaction of CS and GA, which significantly improved the solubility of CS. The UV absorbance spectrum exhibited an obvious characteristic peak, demonstrating the successful preparation of CS-GA (Figure 1B). Aldehyde-modified HA (HA-ALD) was synthesized by oxidizing HA with periodate. H-NMR data further confirmed the successful synthesis of the CS-GA and HA-ALD, as shown in Supplementary Figures S2 and S3. We then added the PDA@HAP into the HA-ALD with ultrasonic stirring. We successfully synthesized the injective hydrogel by a Schiff-base reaction between the aldehyde-modified HA-ALD and the amino group of CS-GA. It provides a smart strategy to fill irregular defects and promote tissue healing (Pan et al., 2020; Zhang et al., 2022a). The sol-to-gel transformation process is shown in Figure 1A The successful synthesis of hydrogels occurred after the two solutions were homogeneously mixed at a volume ratio of 1:1 for 10 s. The FTIR spectra were measured to characterize the chemical structure of the composite hydrogel. As shown in Figure 1C new carbonyl peak of the acyl hydrazone bond appeared at 1662 cm^-1^, indicating the Schiff binding between CS-GA and HA-ALD. The FTIR spectra of the CGH/PDA@HAP hydrogel also showed characteristic peaks of PDA@HAP at 565, 603, and 1033 cm^-1^, demonstrating the successful introduction of PDA@HAP. Further, XRD was performed to analyze the composition of hydrogels. As shown in Figure 1D, the diffraction peak intensities of PDA@HAP were weaker than those of HAP, which indicate that the PDA was coated on the surface of HAP. The main peak of the CGH/PDA@HAP hydrogel is matched to that of HAP, which proves that HAP was present in the hydrogel.

Since the swelling ratio of materials is closely related to the behavior of cells and the effect of tissue healing, the swelling profiles of the CGH and CGH/PDA@HAP hydrogels were evaluated. As shown in Figure 1E, the freeze-dried hydrogels were quickly swelled at the first 1 h, then reached an equilibrium state. Both of the hydrogels exhibited a favorable ability of water uptake. In particular, the swelling ratio decreased through PDA@HAP introducing, proving that PDA grafting is an effective method to make hydrogel stable (Huang et al., 2022). Further, enzymatic degradation assay was performed to evaluate the degradability and stability of the CGH and CGH/PDA@HAP hydrogels in a simulated *in vivo* environment. As show in Figure 1F, the CGH and CGH/PDA@HAP hydrogels could degrade in the hyaluronidase solution, indicating the degradability of them. The long-term application of HA remains challenge due to the enzymatic degradation mediated by tissue (Ito et al., 2007). Previous study has been proved that HA was completely degraded in a hyaluronidase solution with a concentration of 10U/mL for 5 days (Lin et al., 2015). The results in this study indicated that the CGH and CGH/PDA@HAP hydrogels remains more than 25% of residual weight on 5 days even in a relative high concentration of hyaluronidase solution (900U/mL). It can be attributed to that the Schiff-base triggers the crosslinking reaction, thus prolonging the degradation time (Yuan et al., 2017). This is consistent with previous study (Ma et al., 2021).

**FIGURE 2:**
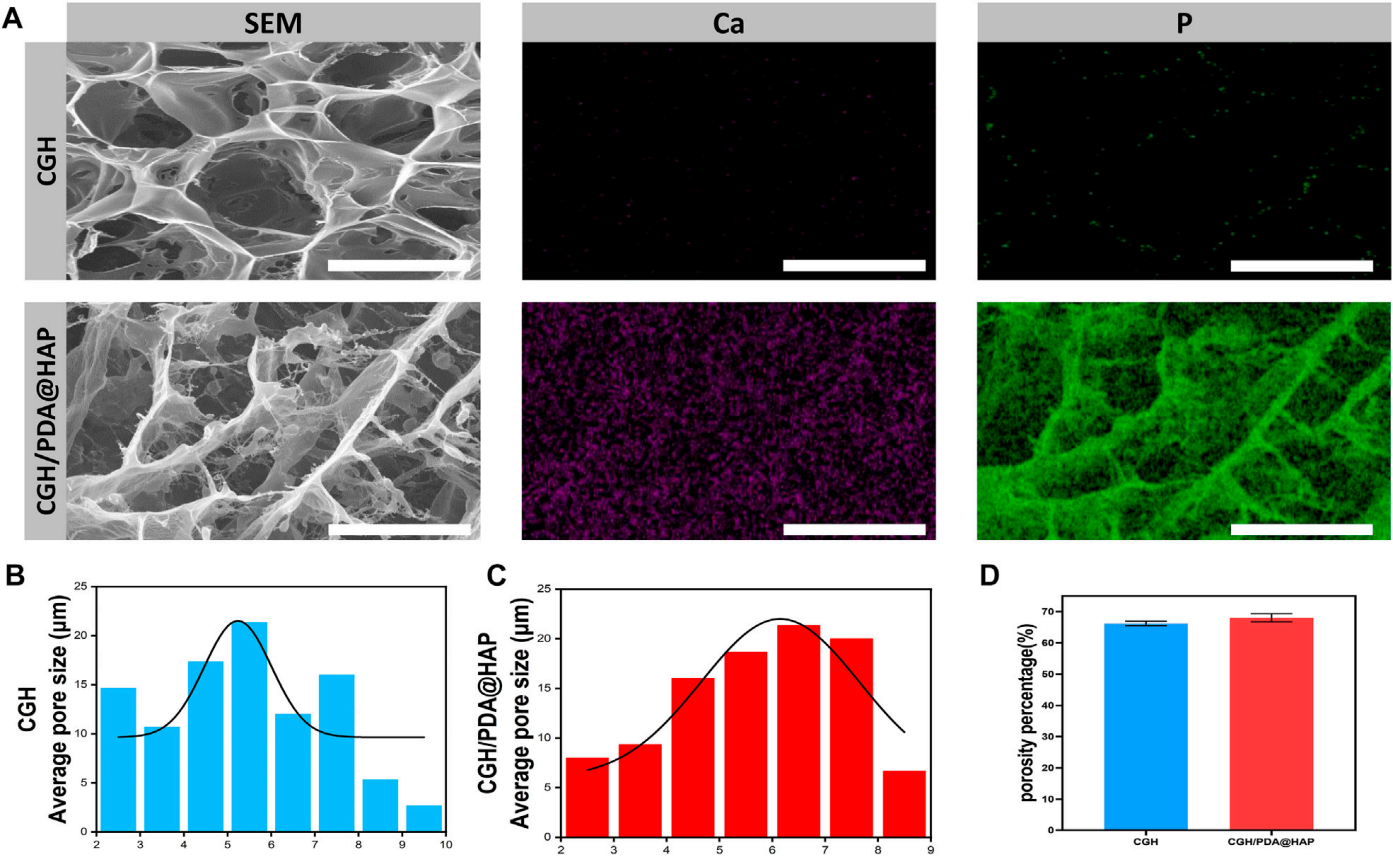
Cyro-SEM observation of the CGH and CGH/PDA@HAP hydrogels. **(A)** SEM images and elemental mapping. **(B)** Average pore size of the CGH hydrogel. **(C)** Average pore size of the CGH/PDA@HAP hydrogel. **(D)** Porosity percentage of the CGH and CGH/PDA@HAP hydrogels. Scale bar: 2.5 μm.

The microstructure of the CGH/PDA@HAP hydrogel was also observed by Cyro-SEM. As shown in Figure 2A, the CGH and CGH/PDA@HAP hydrogel had uniform porous microstructures, indicating the three-dimensional mesh structure of these hydrogels. As shown in Figures 2B–D, the addition of PDA@HAP has no obvious effect on the pore size and porosity percentage of the hydrogel. In particular, the interconnected porous structures could mimic the histological morphology of the native bone, thus increasing the osteo-conductivity (Zhao et al., 2020). This is essential to promote proliferation and differentiation of BMSC (Yi et al., 2016). Moreover, elemental mapping (Figure 2A) demonstrated that the distribution of HAP was evenly on the CGH/PDA@HAP hydrogel through PDA modification, thus overcoming the disadvantage of aggregation (Gao et al., 2022).

### 3.2 Characterization of the CGH/PDA@HAP hydrogel

**FIGURE 3:**
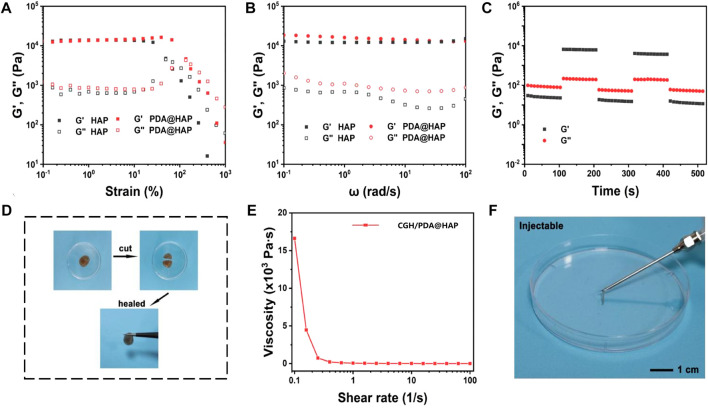
Characterization of the CGH/PDA@HAP hydrogel. **(A)** Rheological strain sweeps. **(B)** Rheological frequency sweeps. **(C)** Step strain sweeps. **(D)** Optical images of the self-healing phenomenon. **(E)** Viscosity as a function of shear rate. **(F)** Optical images of the injectable property of the hydrogel.

The oscillatory rheology of the CGH/PDA@HAP hydrogel was characterized to further investigate the stability and mechanical behaviors. First, we prepared the nano-HAP–loaded CGH (CGH/HAP) hydrogel as a control. As shown in Figure 3A, The CGH/HAP and CGH/PDA@HAP hydrogels exhibited a similar elastic modulus, suggesting that the introduction of PDA had little effect on the mechanical strength. As shown in Figure 3B, the storage modulus (G′) was consistently larger than the loss modulus (G″) when the angular frequency ranged from 0.1 to 100 rad/s, confirming the elastic behavior of the CGH/HAP and CGH/PDA@HAP hydrogels. Moreover, rheological recovery and macroscopic self-healing behavior tests were conducted to estimate the self-healing properties of the CGH/PDA@HAP hydrogel. Based on the strain amplitude sweep measurement, no obvious change of the modulus was observed even after five cycles, demonstrating the outstanding self-healing ability of the CGH/PDA@HAP hydrogel (Figure 3C). We also cut the CGH/PDA@HAP hydrogel into two parts, which healed within a short time (Figure 3D), further indicating the self-healing of the CGH/PDA@HAP hydrogel from a macro perspective.

The dependence of viscosity on shear rate was explored to investigate the injectability of CGH/PDA@HAP hydrogel. As shown in Figure 3E, the viscosity of the hydrogel decreased with the shear rate increased, confirming the CGH/PDA@HAP had the ability to shear thinning. Therefore, the CGH/PDA@HAP hydrogel can be extruded through a syringe and maintain a stable gel state after removing the shear stress, further demonstrating the injectability of the CGH/PDA@HAP hydrogel.

### 3.3 Biocompatibility evaluation

**FIGURE 4:**
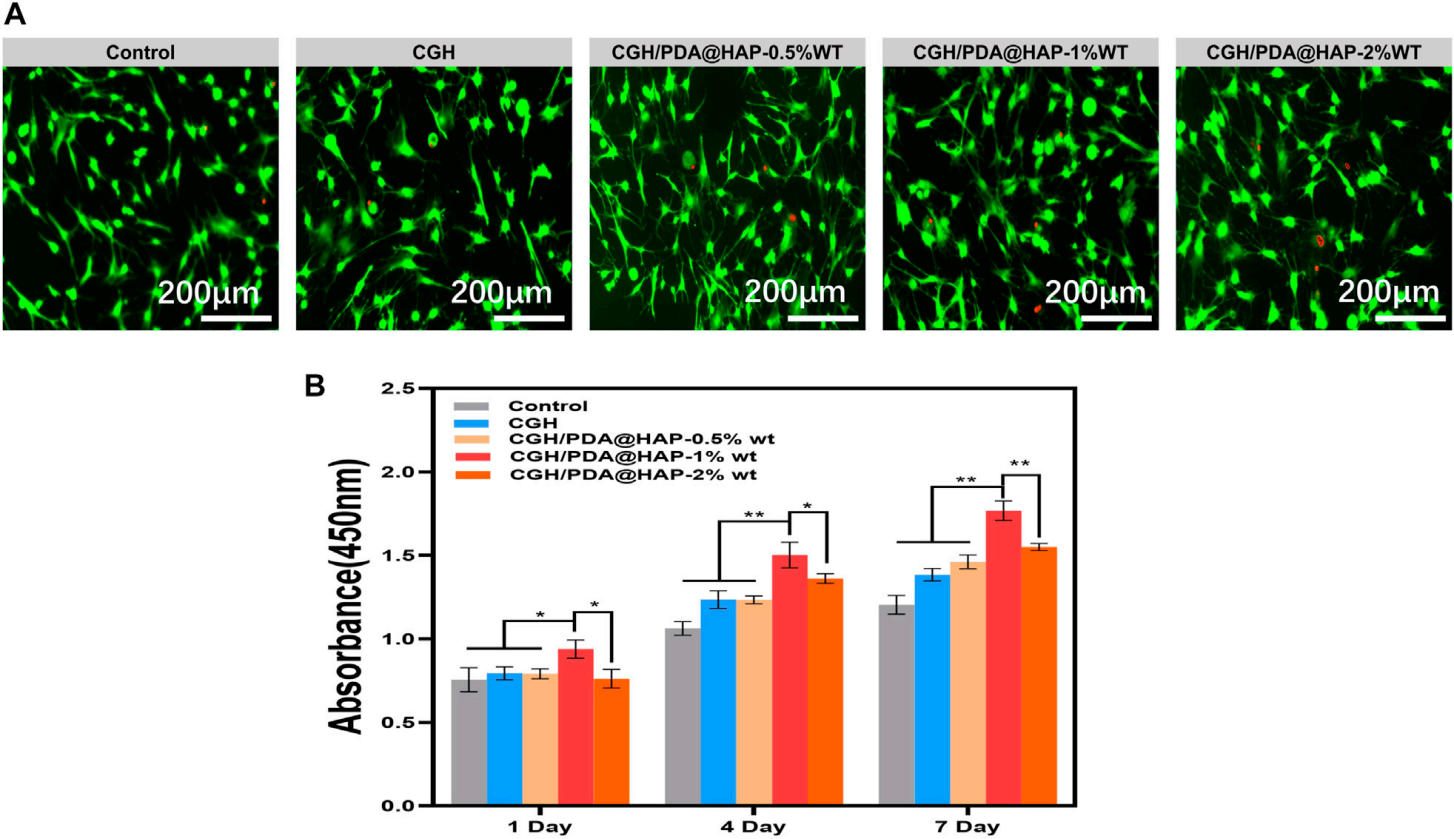
Biocompatibility of CGH and CGH/PDA@HAP hydrogel. **(A)** Live/Dead cell staining. **(B)** CCK-8 assay. (*indicates significant differences; *p < 0.05, **p < 0.01).

To determine whether the concentration of the PDA@HAP in hydrogel could affect the viability of BMSC, three hydrogels containing different mass fractions (0.5% wt, 1% wt and 2% wt) were prepared. As shown in Figure 4A, most of the BMSCs survived and were capable of migration and adhesion in each group, suggesting that the hydrogel scaffolds have minimal cytotoxicity. Notably, BMSCs cultured with the hydrogel containing 1% wt PDA@HAP exhibited higher metabolic rates than the other groups (Figure 4B). Thus, the CGH/PDA@HAP-1%wt was selected for the subsequent studies.

It is generally acknowledged that the advantage of the three-dimensional porous structure of a hydrogel is to guide the orderly migration and proliferation of cells (Zhang et al., 2021c; Zhou et al., 2022). Compared with traditional two-dimensional culture, this three-dimensional culture structure can establish favorable conditions for the growth of cells in a given space, which lays a good foundation for osteogenic differentiation (Shanbhag et al., 2021; Xu et al., 2021; Zhu et al., 2022). As reported in previous studies, a PDA-modified material has an increased surface positive charge, which makes it easier to bind to integrin receptors on the surface of cell membranes and attract cells for attachment on the material (Yu et al., 2021; Im et al., 2022). Moreover, the hydrophilicity of the material itself increases after PDA grafting, which offers another advantage to promote cell attachment (Wu et al., 2019; Zhang et al., 2020). Given these properties, the CGH/PDA@HAP hydrogel was verified to be able to support BMSC adhesion and proliferation, which are key factors contributing to osteogenesis.

### 3.4 Osteogenic differentiation effect of the CGH/PDA@HAP hydrogel *in vitro*

**FIGURE 5:**
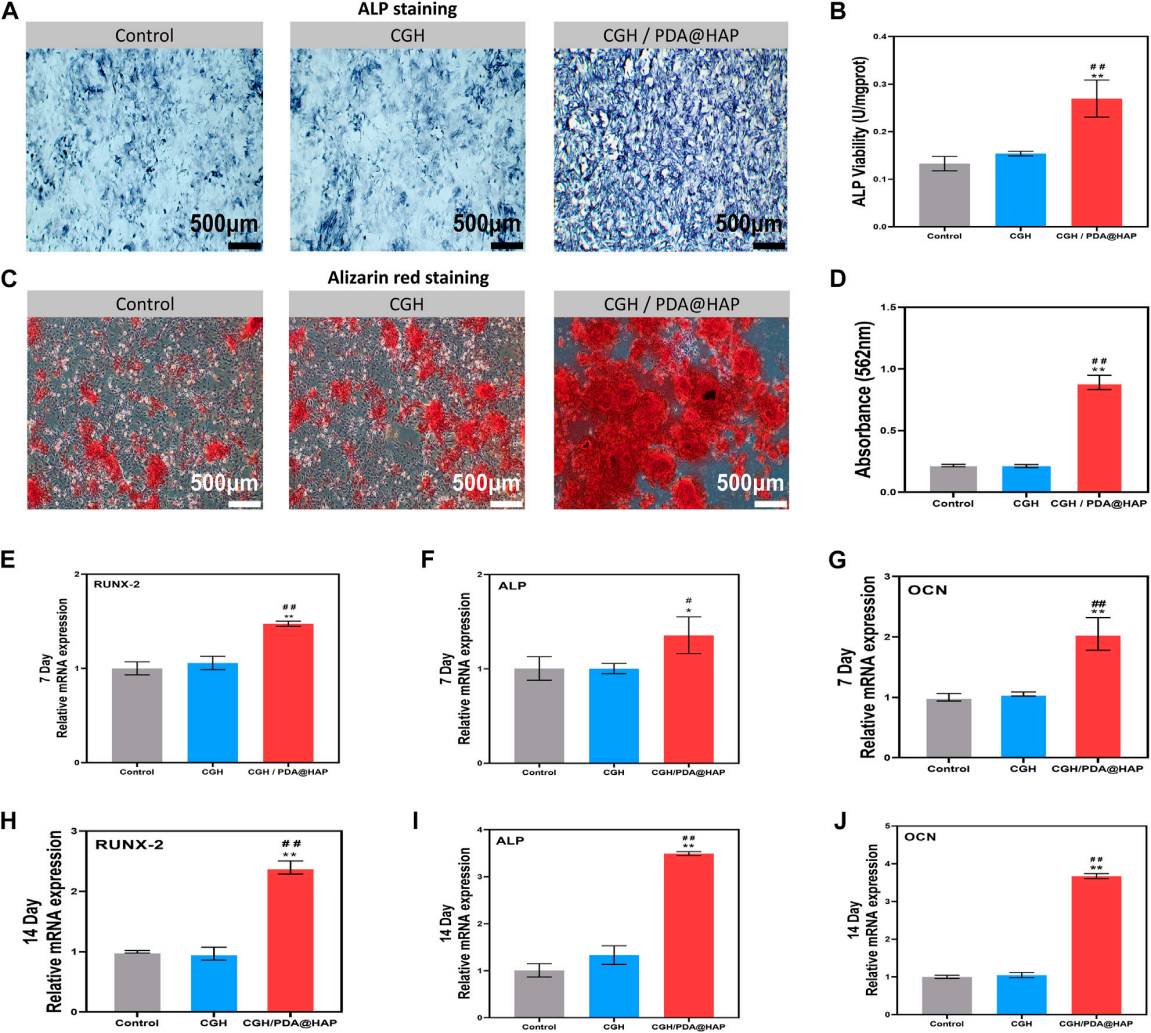
Osteogenic differentiation of BMSCs. **(A)** ALP staining of BMSCs cultured in osteogenic medium supplemented with extracts of various materials for 14 days. **(B)** Quantification of ALP activity of BMSCs cultured in osteogenic medium supplemented with extracts of various materials. **(C)** ARS assay of BMSCs cultured with osteogenic medium supplemented with extracts of various materials for 21 days. **(D)** Quantitative analysis of **(C)**. **(E,F and G)** Expression of osteoblastic genes of BMSCs after culture for 7 days. **(H,I and J)** Expression of osteoblastic genes of BMSCs cultured for 14 days. (*indicates significant differences compared with the control group and # indicates significant differences compared with the CGH group; *p < 0.05, **p < 0.01, #*p* < 0.05, ##*p* < 0.01).

To explore the osteogenic differentiation effect of the CGH/PDA@HAP hydrogel on BMSCs, ALP activity assessment and ARS were performed. As shown in Figures 5A, B, ALP activity was significantly increased in the CGH/PDA@HAP group compared with that of the control and CGH groups when BMSCs were treated for 14 days. After 21 days of treatment, orange-red calcium nodules formed in the extracellular matrix of BMSCs in all groups. However, the number and area of orange-red nodules in the CGH/PDA@HAP group were significantly higher than those in the control and CGH groups (Figure 5C). Corresponding quantitative analysis of ARS demonstrated the same trend and was consistent with the staining results (Figure 5D). It is generally acknowledged that the hydrogel scaffolds are with the remarkable drug loading characteristics and capable to form a slow-release effect in the injured site, ensuring a long-term and effective treatment to promote tissue healing (Zhang et al., 2021b; Wang et al., 2023). In this study, Ca^2+^, as well as PO_4_^3-^, were confirmed to be released from the CGH/PDA@HAP hydrogel (Supplementary Figure S4). These results may reflect the fact that the PDA@HAP in the hydrogel can continuously release Ca^2+^, which can provide a favorable chemical environment for the differentiation of BMSCs into osteoblasts (Ciobanu et al., 2018).

To further evaluate the osteogenic differentiation effect of CGH/PDA@HAP, RT-qPCR was employed to detect the expression of several osteogenesis-related genes, including *Runx2, Alp*, and *Ocn.* As shown in Figures 5E–J, the expression of these genes in the CGH/PDA@HAP group was notably upregulated compared with that in the control and CGH groups, which was consistent with the staining results summarized above. Although HAP can improve the osteogenic conduction and induction of a hydrogel scaffold, its aggregation may cause a high ion concentration in the microenvironment, thereby impairing the proliferation and osteogenic differentiation of BMSCs (Pan et al., 2020; Xu et al., 2020). Therefore, we adopted the strategy of modifying HAP with PDA to evenly distribute the HAP in the hydrogel, preventing the production of excessively high concentrations of ions to provide a favorable condition for osteoblast differentiation (Gao et al., 2022). Moreover, Ca^2+^ can further promote the osteogenic differentiation of BMSCs through the activation of calcium channels on the surface of BMSCs by endogenous electrical signals, thus increasing the intracellular Ca^2+^ concentration (Ciobanu et al., 2018). These results demonstrated that the effect of inducing osteogenic differentiation of BMSC was enhanced when introducing PDA@HAP into the hydrogel, and the expression of osteogenic-related genes was upregulated, especially at 14 days. Moreover, there was no inhibition of cell proliferation and differentiation due to HAP aggregation.

### 3.5 Effect of the CGH/PDA@HAP hydrogel on bone healing *in vivo*

**FIGURE 6:**
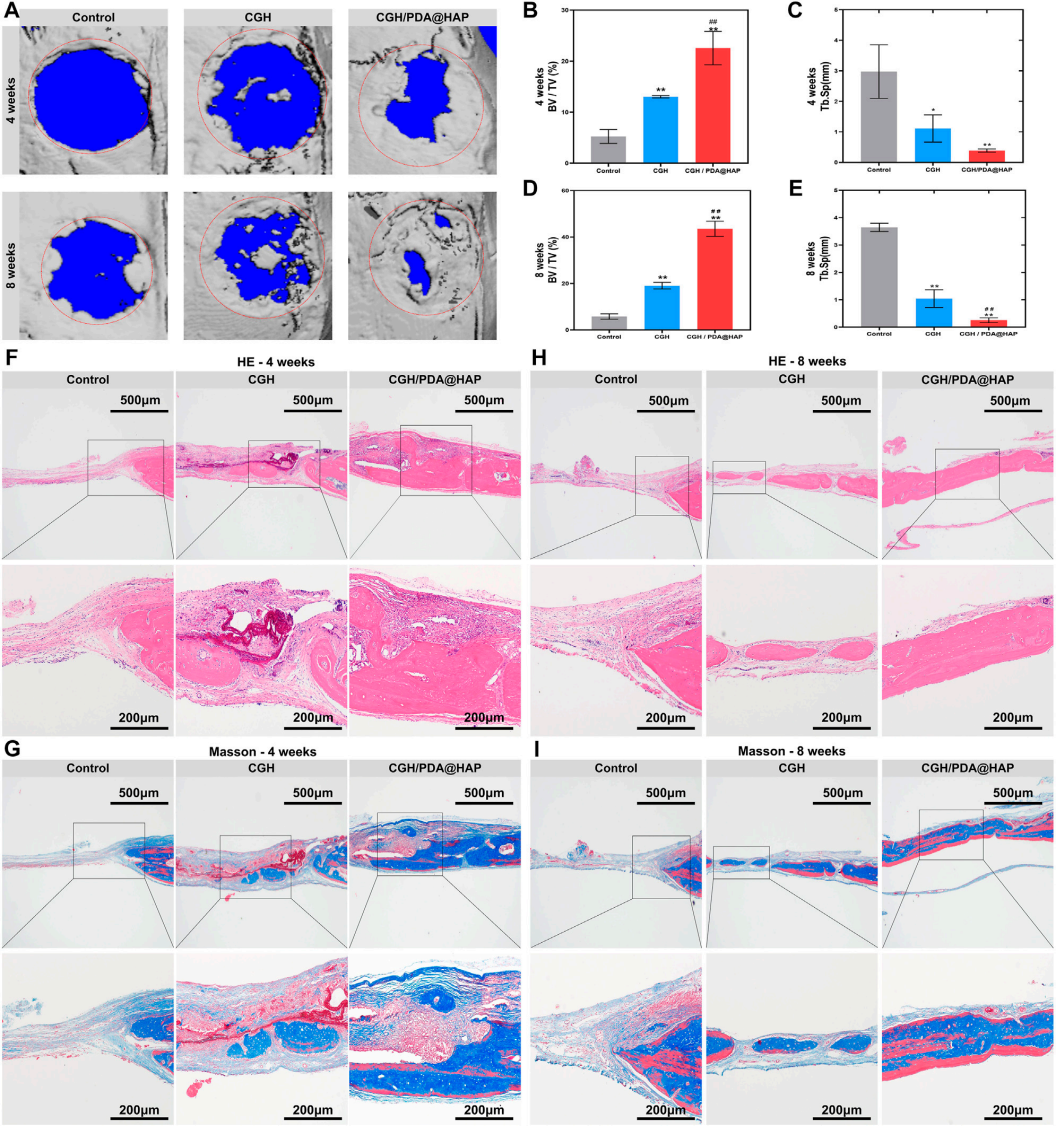
The CGH/PDA@HAP hydrogel promotes bone regeneration *in vivo*. **(A)** Three-dimensional reconstructed micro-CT images of rat cranial bone defects at 4 and 8 weeks after injecting hydrogel materials. Red circles illustrate the original defect area and blue parts in the images represent the background. Gray areas in red circles indicate new bone formation. **(B)** BV/TV of the defect area of each group at 4 weeks post-surgery. **(C)** Tb. Sp of the newly formed bone of each group at 4 weeks post-surgery. **(D)** BV/TV of the defect area of each group at 8 weeks post-surgery. **(E)** Tb. Sp of the newly formed bone of each group at 8 weeks post-surgery. **(F)** HE staining images of each group at 4 weeks post-surgery. **(G)** Masson’s trichrome staining images of each group at 4 weeks post-surgery. **(H)** HE staining images of each group at 8 weeks post-surgery. **(I)** Masson’s trichrome staining images of each group at 8 weeks post-surgery. (*indicates significant differences compared with the control group and # indicates significant differences compared with the CGH group; *p < 0.05, **p < 0.01, ##*p* < 0.01).

To evaluate the bone healing effect of the CGH/PDA@HAP hydrogel *in vivo*, a 5-mm-diameter critical cranial defect was created in SD rats to study the osteogenic effect. At 4 weeks and 8 weeks post-surgery, micro-CT was employed to assess the healing of the cranium defect. As shown in Figure 6A, bone healing in the CGH/PDA@HAP group was more obvious compared to that observed in the control and CGH groups at both 4 and 8 weeks post-surgery. At 8 weeks after implantation, the CGH/PDA@HAP hydrogel induced significant new bone formation both in the periphery and in the center of the defect. Moreover, the BV/TV was remarkably increased in the CGH/PDA@HAP group compared with that of the control and CGH groups, whereas the opposite result was found for the Tb. Sp measurement (Figures 6B–E). These findings suggested that the CGH/PDA@HAP hydrogel has improved osteogenic effects *in vivo*, consistent with the results found *in vitro*.

This effect can be attributed to the fact that the differentiation of BMSCs into osteoblasts is closely related to the structure of scaffold materials (Zhang et al., 2021c). The three-dimensional porous structure of the hydrogel can not only recruit more cells to the defect area, but its morphological regulatory function also plays an essential role in the osteogenic differentiation of BMSCs (Xu et al., 2021; Zhu et al., 2022). The *in vivo* three-dimensional culture conditions created by the hydrogel not only facilitate the migration and proliferation of BMSCs through the paracrine secretion of exosomes via activation of the HMGB1/AKT pathway, but also synergize with osteogenically active substances in the system to accelerate osteogenic differentiation (Matsumoto et al., 2011; Oliveros et al., 2021; Ren et al., 2021; Tavakol et al., 2021). More importantly, the catechol group on the surface of PDA-modified scaffolds can effectively anchor calcium ions in HAP, which stabilizes the local calcium concentration and induces the osteogenic differentiation of BMSCs (Gao et al., 2022). On this basis, the formation of hydroxyapatite on the scaffold surface can be promoted to accelerate the process of osseointegration (Matsumoto et al., 2011; Wu et al., 2011; Wu et al., 2019; Zhang et al., 2021a).

Furthermore, HE staining and Masson’s trichrome staining were used to verify the effect of the CGH/PDA@HAP hydrogel on bone defect healing at the histology level. As shown in Figures 6F,H large amount of newly regenerated bone was induced from the margin to the central area of the defect in the CGH/PDA@HAP group, whereas new bone formation was hardly detected in the control group. Masson’s trichrome staining images showed the same tendency as found for the HE staining results (Figures 6G,I). In addition, no obvious pathological changes were observed in the HE-staining images of the major organs, including the heart, liver, spleen, lung, and kidney, suggesting that the CGH/PDA@HAP hydrogel has good biological safety *in vivo* (Supplementary Figure S5). The Cyro-SEM images further proved that the CGH/PDA@HAP hydrogel has a three-dimensional porous structure, which is a key feature to endow the hydrogel with excellent osteogenic conduction for directing BMSC migration toward the center of the bone defect. Moreover, PDA enhanced the bioactivity of the hydrogel surface and made it easier to recruit cells, laying a solid foundation for bone regeneration (Gao et al., 2022). Significantly, the HAP in the hydrogel can effectively induce the osteogenic differentiation of BMSCs, promoting mineralization of the extracellular matrix to form mature new bone (Zhao et al., 2020). These findings are thus consistent with the results of the *in vitro* experiments. In summary, the CGH/PDA@HAP hydrogel recruited cells to the center of the defect and regulated the behavior of cells in a programmed manner at both the internal morphology and the active substance, thus achieving a satisfactory therapeutic effect of bone healing.

### 3.6 Antibacterial effect *in vitro*

**FIGURE 7:**
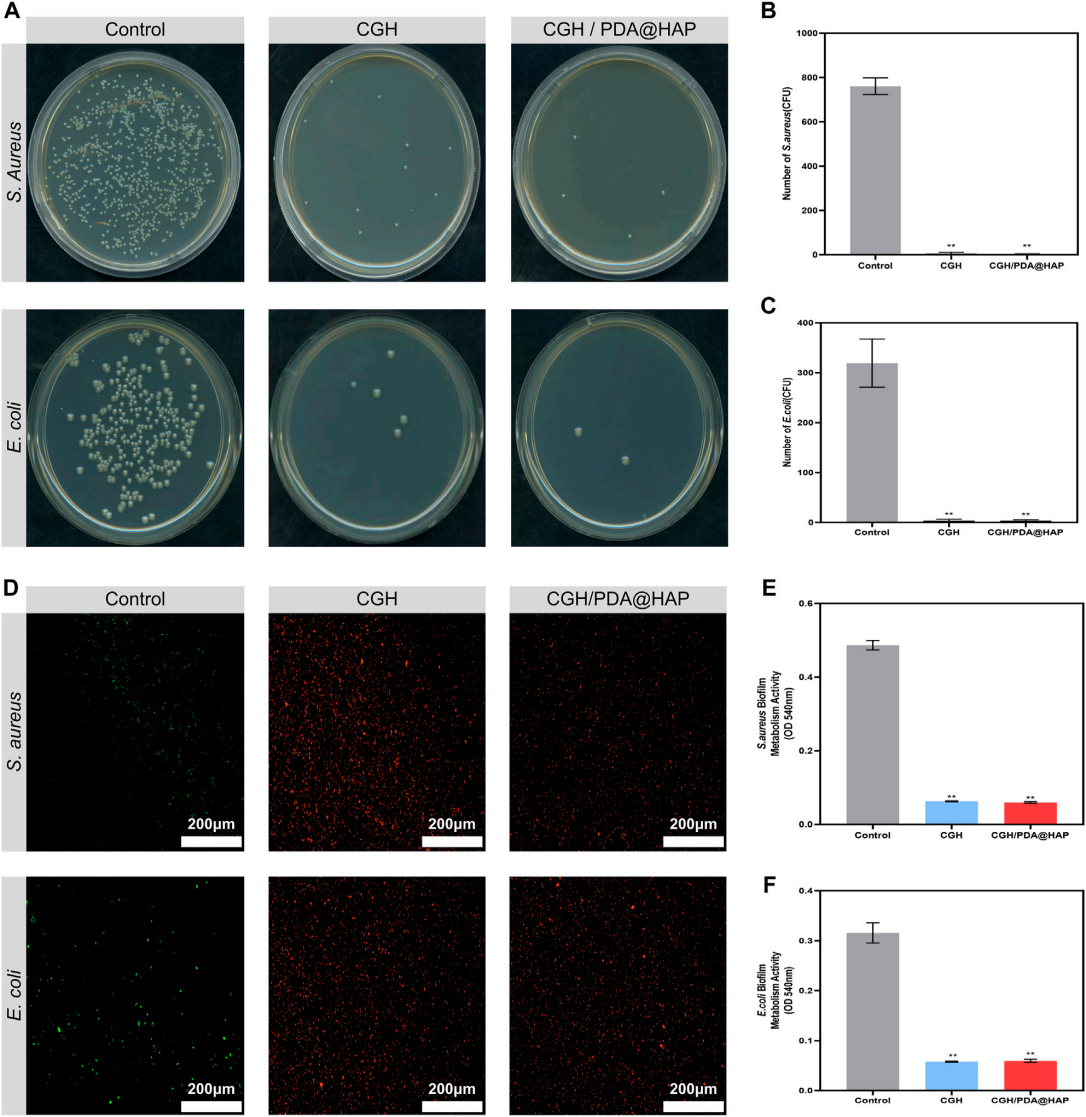
Antibacterial properties of the CGH/PDA@HAP and CGH hydrogels against *Staphylococcus aureus* and *Escherichia coli*. **(A)** Typical images of *Staphylococcus aureus* and *Escherichia coli* bacterial colonies after culture with CGH/PDA@HAP and CGH hydrogels. **(B,C)** Quantitative analysis of **(A)**. **(D)** Live/dead bacteria staining to evaluate the capability of CGH/PDA@HAP and CGH hydrogels to prevent adhesion of *Staphylococcus aureus* and *Escherichia coli.* **(E,F)** MTT assay to assess the effects of CGH/PDA@HAP and CGH hydrogels on the metabolic activity of *Staphylococcus aureus* and *Escherichia coli* biofilms. (*indicates significant differences compared with the control group; **p < 0.01).

*S. aureus* and *E. coli* were used to study the antibacterial effect of the CGH and CGH/PDA@HAP hydrogels on Gram-negative and Gram-positive bacteria, respectively. To evaluate the inhibiting effect of the CGH and CGH/PDA@HAP hydrogels on planktonic bacteria, time-kill curves were constructed. As shown in Supplementary Figure S7, the growth curves of *S. aureus* and *E. coli* exhibited an increasing trend within 12 h in the control group, whereas growth was maintained at a stable level in the CGH and CGH/PDA@HAP groups. To further investigate these effects, the CFU count was quantified. Accordingly, colony formation was significantly inhibited when the bacteria were cultured with CGH and CGH/PDA@HAP hydrogels (Figure 7A). Quantitative analysis demonstrated that the inhibitory rate of the CGH hydrogel against *S. aureus* and *E. coli* was above 90% (Figures 7B, C). To further investigate the inhibition of *S. aureus* and *E. coli* biofilm formation, bacteria live and dead staining was used, in which live bacteria are stained with green fluorescence while dead bacteria are stained red. As shown in Figure 7D, *S. aureus* and *E. coli* in the CGH and CGH/PDA@HAP groups emitted a strong red signal, indicating that most of the bacteria had been killed on the hydrogels. Moreover, the MTT assay showed that the metabolic activity of *S. aureus* and *E. coli* biofilms significantly decreased in both the CGH and CGH/PDA@HAP groups compared with that of the control group (Figures 7E, F).

*Staphylococcus aureus* is considered the main pathogen of postoperative infection in open bone defect cases, which is attributed to the fact that it can induce the differentiation of osteoclasts via the secretion of proinflammatory factors and proteins, either directly or indirectly (Tong et al., 2022). Moreover, intense inflammatory reactions initiated by infection lead to the release of inflammatory factors, which inevitably increases apoptosis and inhibits the osteogenic differentiation of BMSCs (Xu et al., 2016). Our results demonstrated that the CGH/PDA@HAP hydrogel represents a scaffold with broad-spectrum antibacterial activity that could inhibit *S. aureus*, thereby showing potential to prevent infection and effectively avoiding the negative effects of inflammation on bone regeneration.

Notably, GA can penetrate the bacterial cell wall to change osmotic pressure and inhibit the bacterial respiratory chain, thus reducing the drug resistance (Shi et al., 2021; Wei et al., 2023). A previous study demonstrated that GA can effectively decrease metabolic activity and destroy the biofilms of *S. aureus* and *E. coli* (Sun et al., 2022)*.* In this study, the CGH and CGH/PDA@HAP hydrogels successfully inhibited *S. aureus* and *E. coli,* preventing bacterial colonization and biofilm formation, and reducing metabolic activity. Therefore, the inherent antibacterial properties of hydrogel scaffolds can be improved through GA grafting even without loading antibacterial substances, which overcomes the lack of antibacterial properties of traditional bone scaffolds.

## 4 Conclusion

We developed a mussel-inspired mineral CGH/PDA@HAP hydrogel through the assembly of PDA@HAP, CS, GA, and HA, which exhibited desirable properties, including a porous structure, tunable mechanical property, injectability, as well as self-healing ability. It is expected that PDA enhances the bioactivity of the hydrogel and plays a positive role in bone formation in collaboration with HAP. Moreover, GA modification was used to improve the antibacterial properties of the hydrogel so as to promote bone regeneration while preventing infection. Based on this concept, we have proven that the CGH/PDA@HAP hydrogel has superior performance for promoting the adhesion, proliferation, and osteogenic differentiation of BMSCs *in vitro*. Notably, animal experiments further proved that the CGH/PDA@HAP hydrogel could meet the demand of promoting bone tissue regeneration and reconstruction when the defect reaches a certain critical size that prevents regeneration. In addition, GA grafting greatly improved the antibacterial performance of the hydrogel system, which inhibited the growth, adhesion, colonization, and biofilm formation of *S. aureus* and *E. coli in vitro*. In conclusion, our study provides a new strategy for treating open bone defects and preventing the occurrence of infection during bone healing.

## Data Availability

The original contributions presented in the study are included in the article/Supplementary Material, further inquiries can be directed to the corresponding authors.

## References

[B1] Asha A. B. Chen Y. Narain R. (2022). Bioinspired dopamine and zwitterionic polymers for non-fouling surface engineering. Chem. Soc. Rev. 50, 11668–11683. 10.1039/d1cs00658d 34477190

[B2] Chen C. Zhou P. Huang C. Zeng R. Yang L. Han Z. (2021). Photothermal-promoted multi-functional dual network polysaccharide hydrogel adhesive for infected and susceptible wound healing. Carbohydr. Polym. 273, 118557. 10.1016/j.carbpol.2021.118557 34560968

[B3] Chen Y. Zhang F. Fu Q. Liu Y. Wang Z. Qi N. (2016). *In vitro* proliferation and osteogenic differentiation of human dental pulp stem cells in injectable thermo-sensitive chitosan/β-glycerophosphate/hydroxyapatite hydrogel. J. Biomater. Appl. 31, 317–327. 10.1177/0885328216661566 27496540

[B4] Ciobanu F. Golzio M. Kovacs E. Teissié J. (2018). Control by low levels of calcium of mammalian cell membrane electropermeabilization. J. Membr. Biol. 251, 221–228. 10.1007/s00232-017-9981-y 28823021

[B5] Cui J. Yan Y. Such G. K. Liang K. Ochs C. J. Postma A. (2012). Immobilization and intracellular delivery of an anticancer drug using mussel-inspired polydopamine capsules. Biomacromolecules 13, 2225–2228. 10.1021/bm300835r 22792863

[B6] Gao F. Xu Z. Liang Q. Liu B. Li H. Wu Y. (2018). Direct 3D printing of high strength biohybrid gradient hydrogel scaffolds for efficient repair of osteochondral defect. Adv. Funct. Mat. 28, 1706644. 10.1002/adfm.201706644

[B7] Gao F. Zeng D. Liu H. Qin R. Zhang J. Chen Y. (2022). Porous cellulose microspheres coated in one step with a polydopamine suspension of hydroxyapatite for bone tissue engineering. Cellulose 29, 1955–1967. 10.1007/s10570-021-04395-4

[B8] Guan L. Yan S. Liu X. Li X. Gao G. (2019). Wearable strain sensors based on casein-driven tough, adhesive and anti-freezing hydrogels for monitoring human-motion. J. Mat. Chem. B 7, 5230–5236. 10.1039/c9tb01340g 31378805

[B9] Han L. Li P. Tang P. Wang X. Zhou T. Wang K. (2019). Mussel-inspired cryogels for promoting wound regeneration through photobiostimulation, modulating inflammatory responses and suppressing bacterial invasion. Nanoscale 29, 15846–15861. 10.1039/c9nr03095f 31289795

[B10] Huang B. Chen M. Tian J. Zhang Y. Dai Z. Li J. (2022). Oxygen-carrying and antibacterial fluorinated nano-hydroxyapatite incorporated hydrogels for enhanced bone regeneration. Adv. Healthc. Mat. 11, 2102540. 10.1002/adhm.202102540 35166460

[B11] Im S. Choe G. Seok J. M. Yeo S. J. Lee J. H. Kim W. D. (2022). An osteogenic bioink composed of alginate, cellulose nanofibrils, and polydopamine nanoparticles for 3D bioprinting and bone tissue engineering. Int. J. Biol. Macromol. 30, 520–529. 10.1016/j.ijbiomac.2022.02.012 35217077

[B12] Ito T. Yeo Y. Highley C. B. Bellas E. Benitez C. A. Kohane D. S. (2007). The prevention of peritoneal adhesions by *in situ* cross-linking hydrogels of hyaluronic acid and cellulose derivatives. Biomaterials 28, 975–983. 10.1016/j.biomaterials.2006.10.021 17109954 PMC1859847

[B13] Jiang L. Li Y. Xiong C. Su S. Ding H. (2017). Preparation and properties of bamboo fiber/nano-hydroxyapatite/poly(lactic-co-glycolic) composite scaffold for bone tissue engineering. Acs. Appl. Mat. 9, 4890–4897. 10.1021/acsami.6b15032 28084718

[B14] Lee J. S. Baek S. D. Venkatesan J. Bhatnagar I. Chang H. K. Kim H. T. (2014). *In vivo* study of chitosan-natural nano hydroxyapatite scaffolds for bone tissue regeneration. Int. J. Biol. Macromol. 67, 360–366. 10.1016/j.ijbiomac.2014.03.053 24705167

[B15] Leng Q. Chen L. Lv Y. (2020). RNA-Based scaffolds for bone regeneration: Application and mechanisms of mRNA, miRNA and siRNA. Theranostics 10, 3190–3205. 10.7150/thno.42640 32194862 PMC7053199

[B16] Li Y. Yang L. Hou Y. Zhang Z. Chen M. Wang M. (2022). Polydopamine-mediated graphene oxide and nanohydroxyapatite-incorporated conductive scaffold with an immunomodulatory ability accelerates periodontal bone regeneration in diabetes. Bioact. Mat. 18, 213–227. 10.1016/j.bioactmat.2022.03.021 PMC8961429 35387166

[B17] Lin C. Peng H. Chen M. Sun J. Liu T. Chen M. (2015). *In situ* forming hydrogel composed of hyaluronate and polygalacturonic acid for prevention of peridural fibrosis. J. Mat. Sci. Mat. Med. 26, 168. 10.1007/s10856-015-5478-3 25791456

[B18] Liu L. Shi J. Sun X. Zhang Y. Qin J. Peng S. (2022). Thermo-responsive hydrogel-supported antibacterial material with persistent photocatalytic activity for continuous sterilization and wound healing. Compos. Part. B-Eng. 229, 109459. 10.1016/j.compositesb.2021.109459

[B19] Liu Z. Tang M. Zhao J. Chai R. Kang J. (2018). Looking into the future: Toward advanced 3D biomaterials for stem-cell-based regenerative medicine. Adv. Mat. 30, e1705388. 10.1002/adma.201705388 29450919

[B20] Lüthje F. L. Jensen L. K. Jensen H. E. Skovgaard K. (2020). The inflammatory response to bone infection-a review based on animal models and human patients. APMIS 128, 275–286. 10.1111/apm.13027 31976582

[B21] Ma X. Yang R. Kanaparedu P. C. Chi B. (2021). Injectable hyaluronic acid/poly(γ-glutamic acid) hydrogel with step-by-step tunable properties for soft tissue engineering. Chin. J. Polym. Sci. 39, 957–965. 10.1007/s10118-021-2558-3

[B22] Matsumoto T. Mizuno A. Kashiwagi M. Yoshida S. S. Sasaki J. I. Nakano T. (2011). Cell-based fabrication of organic/inorganic composite gel material. Materials 4, 327–338. 10.3390/ma4010327 28879992 PMC5448484

[B23] Oliveros A. L. Kingham P. J. Lammi M. J. Wiberg M. Kelk P. (2021). Three-dimensional osteogenic differentiation of bone marrow mesenchymal stem cells promotes matrix metallopeptidase 13 (MMP13) expression in type I collagen hydrogels. Int. J. Mol. Sci. 22, 13594. 10.3390/ijms222413594 34948393 PMC8706974

[B24] Pan Y. Zhao Y. Kuang R. Liu H. Sun D. Mao T. (2020). Injectable hydrogel-loaded nano-hydroxyapatite that improves bone regeneration and alveolar ridge promotion. Mat. Sci. Eng. C 116, 111158. 10.1016/j.msec.2020.111158 32806272

[B25] Park J. Brust T. F. Lee H. J. Lee S. C. Watts V. J. Yeo Y. (2014). Polydopamine-based simple and versatile surface modification of polymeric nano drug carriers. Acs. Nano. 8, 3347–3356. 10.1021/nn405809c 24628245 PMC4107448

[B26] Peng S. Meng H. Ouyang Y. Chang J. (2014). Nanoporous magnetic cellulose-chitosan composite microspheres: Preparation, characterization, and application for Cu(II) adsorption. Ind. Eng. Chem. Res. 53, 2106–2113. 10.1021/ie402855t

[B27] Ren S. Tang X. Liu L. Meng F. Yang X. Li N. (2022). Reinforced blood-derived protein hydrogels enable dual-level regulation of bio-physiochemical microenvironments for personalized bone regeneration with remarkable enhanced efficacy. Nano. Lett. 22, 3904–3913. 10.1021/acs.nanolett.2c00057 35522592

[B28] Ren Y. Zhang H. Wang Y. Du B. Yang J. Liu L. (2021). Hyaluronic acid hydrogel with adjustable stiffness for mesenchymal stem cell 3D culture via related molecular mechanisms to maintain stemness and induce cartilage differentiation. Acs. Appl. Bio. Mat. 4, 2601–2613. 10.1021/acsabm.0c01591 35014377

[B29] Sahiner N. Sagbas S. Sahiner M. Silan C. Aktas N. Turk M. (2016). Biocompatible and biodegradable poly(tannic acid) hydrogel with antimicrobial and antioxidant properties. Int. J. Biol. Macromol. 82, 150–159. 10.1016/j.ijbiomac.2015.10.057 26526171

[B30] Shanbhag S. Suliman S. Mohamed-Ahmed S. Kampleitner C. Hassan M. N. Heimel P. (2021). Bone regeneration in rat calvarial defects using dissociated or spheroid mesenchymal stromal cells in scaffold-hydrogel constructs. Stem. Cell. Res. Ther. 12, 575. 10.1186/s13287-021-02642-w 34776000 PMC8591809

[B31] Shi Y. G. Zhang R. R. Zhu C. M. Xu M. F. Gu Q. Ettelaie R. (2021). Antimicrobial mechanism of alkyl gallates against *Escherichia coli* and *Staphylococcus aureus* and its combined effect with electrospun nanofibers on Chinese Taihu icefish preservation. Food. Chem. 346, 128949. 10.1016/j.foodchem.2020.128949 33418419

[B32] Sun C. Zeng X. Zheng S. Wang Y. Li Z. Zhang H. (2022). Bio-adhesive catechol-modified chitosan wound healing hydrogel dressings through glow discharge plasma technique. Chem. Eng. J. 427, 130843. 10.1016/j.cej.2021.130843

[B33] Sun S. Mao L. B. Lei Z. Yu S. H. Cölfen H. (2016). Hydrogels from amorphous calcium carbonate and polyacrylic acid: Bio-inspired materials for “mineral plastics”. Angew. Chem. Int. Ed. Engl. 55, 11765–11769. 10.1002/anie.201602849 27444970

[B34] Tavakol D. N. Bonini F. Tratwal J. Genta M. Brefie-Guth J. Braschler T. (2021). Cryogel-based injectable 3D microcarrier co-culture for support of hematopoietic progenitor niches. Curr. Protoc. 1, e275. 10.1002/cpz1.275 34813179

[B35] Tong Z. Chen Z. Li Z. Xie Z. Zhang H. (2022). Mechanisms of promoting the differentiation and bone resorption function of osteoclasts by *Staphylococcus aureus* infection. Int. J. Med. Microbiol. 312, 151568. 10.1016/j.ijmm.2022.151568 36240531

[B36] Wan Z. Dong Q. Y. Liu Y. S. Zhang X. Zhang P. Lv L. W. (2022). Programmed biomolecule delivery orchestrate programmed biomolecule delivery orchestrate bone tissue regeneration via MSC recruitment and epigenetic modulation. Chem. Eng. J. 438, 135518. 10.1016/j.cej.2022.135518

[B37] Wang F. Liu S. Ren C. Xiang S. Li D. Hao X. (2021b). Construction of hollow polydopamine nanoparticle based drug sustainable release system and its application in bone regeneration. Int. J. Oral. Sci. 13, 27. 10.1038/s41368-021-00132-6 34408132 PMC8373924

[B38] Wang F. Liu Y. Qiu X. Fei H. Liu W. Yuan K. (2021a). Effect of anti-infective reconstituted bone xenograft combined with external fixator on serum CRP and PCT levels and prognosis of patients with bone infection after lower extremity long bone trauma. Evid. Based. Complement. Altern. Med. 30, 5979514. 10.1155/2021/5979514 PMC8423557 34504538

[B39] Wang N. Qi D. Liu L. Zhu Y. Liu H. Zhu S. (2022). Fabrication of *in situ* grown hydroxyapatite nanoparticles modified porous polyetheretherketone matrix composites to promote osteointegration and enhance bone repair. Front. Bioeng. Biotechnol. 10, 831288. 10.3389/fbioe.2022.831288 35295654 PMC8919038

[B40] Wang Y. Zhu L. Wei L. Zhou Y. Yang Y. Zhang L. (2023). A bio-orthogonally functionalized chitosan scaffold with esterase-activatable release for nerve regeneration. Int. J. Biol. Macromol. 229, 146–157. 10.1016/j.ijbiomac.2022.12.113 36528149

[B41] Wei J. Zhu L. Lu Q. Li G. Zhou Y. Yang Y. (2023). Recent progress and applications of poly(beta amino esters)-based biomaterials. J. Control. Release. 354, 337–353. 10.1016/j.jconrel.2023.01.002 36623697

[B42] Wu C. Fan W. Chang J. Xiao Y. (2011). Mussel-inspired porous SiO_2_ scaffolds with improved mineralization and cytocompatibility for drug delivery and bone tissue engineering. J. Mat. Chem. 21, 18300–18307. 10.1039/c1jm12770e

[B43] Wu J. Cao L. Liu Y. Zheng A. Jiao D. Zeng D. (2019). Functionalization of silk fibroin electrospun scaffolds via BMSC affinity peptide grafting through oxidative self-polymerization of dopamine for bone regeneration. Acs. Appl. Mat. Interfaces. 11, 8878–8895. 10.1021/acsami.8b22123 30777748

[B44] Xie L. Liu N. Xiao Y. Liu Y. Yan C. Wang G. (2020). *In vitro* and *in vivo* osteogenesis induced by icariin and bone morphogenetic protein-2: A dynamic observation. Front. Pharmacol. 11, 1058. 10.3389/fphar.2020.01058 32760277 PMC7373825

[B45] Xu H. Wang C. Liu C. Li J. Peng Z. Guo J. (2021). Stem cell-seeded 3D-printed scaffolds combined with self-assembling peptides for bone defect repair. Tissue. Eng. Part. a. 28, 111–124. 10.1089/ten.TEA.2021.0055 34157886

[B46] Xu J. Wang Y. Li J. Zhang X. Geng Y. Huang Y. (2016). IL-12p40 impairs mesenchymal stem cell-mediated bone regeneration via CD4+ T cells. Cell. death. Differ. 23, 1941–1951. 10.1038/cdd.2016.72 27472064 PMC5136484

[B47] Xu L. Bai X. Yang J. Li J. Xing J. Yuan H. (2020). Preparation and characterisation of a gellan gum-based hydrogel enabling osteogenesis and inhibiting *Enterococcus faecalis* . Int. J. Biol. Macromol. 165, 2964–2973. 10.1016/j.ijbiomac.2020.10.083 33086112

[B48] Yang X. Wang B. Sha D. Liu Y. Liu Z. Shi K. (2021). PVA/poly(hexamethylene guanidine)/gallic acid composite hydrogel films and their antibacterial performance. Acs. Appl. Polym. Mat. 3, 3867–3877. 10.1021/acsapm.1c00447

[B49] Yi H. Rehman F. U. Zhao C. Liu B. He N. (2016). Recent advances in nano scaffolds for bone repair. Bone Res. 4, 16050. 10.1038/boneres.2016.50 28018707 PMC5153570

[B50] Yu Q. Zheng Z. Dong X. Cao R. Zhang S. Wu X. (2021). Mussel-inspired hydrogels as tough, self-adhesive and conductive bioelectronics: A review. Soft. Matter. 17, 8786–8804. 10.1039/d1sm00997d 34596200

[B51] Yuan L. Wu Y. Gu Q. El-Hamshary H. El-Newehy M. Mo X. (2017). Injectable photo crosslinked enhanced double-network hydrogels from modified sodium alginate and gelatin. Int. J. Biol. Macromol. 96, 569–577. 10.1016/j.ijbiomac.2016.12.058 28017764

[B52] Zhang C. Wu B. Zhou Y. Zhou F. Liu W. Wang Z. (2020). Mussel-inspired hydrogels: From design principles to promising applications. Chem. Soc. Rev. 49, 3605–3637. 10.1039/c9cs00849g 32393930

[B53] Zhang J. He X. Yu S. Zhu J. Wang H. Tian Z. (2021a). A novel dental adhesive containing Ag/polydopamine-modified HA fillers with both antibacterial and mineralization properties. J. Dent. 111, 103710. 10.1016/j.jdent.2021.103710 34090992

[B54] Zhang J. Liu M. Pei R. (2021c). An *in situ* gelling BMSC-laden collagen/silk fibroin double network hydrogel for cartilage regeneration. Mat. Advan. 2, 4733–4742. 10.1039/d1ma00285f

[B55] Zhang J. Yao K. Wang Y. Zhou Y. Fu Z. Li G. (2021b). Brain-targeted dual site-selective functionalized poly(β-Amino Esters) delivery platform for nerve regeneration. Nano. Lett. 21, 3007–3015. 10.1021/acs.nanolett.1c00175 33797927

[B56] Zhang L. Li J. Chen J. Peng Z. X. Chen J. N. Liu X. (2022b). Enhanced bone regeneration via PHA scaffolds coated with polydopamine-captured BMP2. J. Mat. Chem. B 10, 6214–6227. 10.1039/d2tb01122k 35920210

[B57] Zhang L. Yao K. Wei J. Li G. Lin Y. Zhou Y. (2022a). Convenient *in situ* synthesis of injectable lysine-contained peptide functionalized hydrogels for spinal cord regeneration. Appl. Mat. Today 27, 101506. 10.1016/j.apmt.2022.101506

[B58] Zhao Y. Li Z. Jiang Y. Liu H. Feng Y. Wang Z. (2020). Bioinspired mineral hydrogels as nanocomposite scaffolds for the promotion of osteogenic marker expression and the induction of bone regeneration in osteoporosis. Acta. Biomater. 113, 614–626. 10.1016/j.actbio.2020.06.024 32565370

[B59] Zhou S. Bei Z. Wei J. Yan X. Wen H. Cao Y. (2022). Mussel-inspired injectable chitosan hydrogel modified with catechol for cell adhesion and cartilage defect repair. J. Mat. Chem. B 10, 1019–1030. 10.1039/d1tb02241e 34994756

[B60] Zhou Y. Z. Cao Y. Liu W. Chu C. H. Li Q. L. (2012). Polydopamine-induced tooth remineralization. Acs. Appl. Mat. Interfaces. 4, 6901–6910. 10.1021/am302041b 23176019

[B61] Zhu Y. Ye L. Cai X. Li Z. Fan Y. Yang F. (2022). Icariin-loaded hydrogel regulates bone marrow mesenchymal stem cell chondrogenic differentiation and promotes cartilage repair in osteoarthritis. Front. Bioeng. Biotechnol. 10, 755260. 10.3389/fbioe.2022.755260 35223781 PMC8864219

