# Supplementary material for: Gallic acid-grafted chitosan antibacterial hydrogel incorporated with polydopamine-modified hydroxyapatite for enhancing bone healing
Source: Front Bioeng Biotechnol. 2023 Jun 2;11:1162202. doi: 10.3389/fbioe.2023.1162202 (PMC10273101; doi:10.3389/fbioe.2023.1162202)
Supplement: Supplementary file 1 [file DataSheet1.pdf]

Supplementary Material

Polydopamine-Modified Hydroxyapatite-Incorporated and Gallic Acid-Grafted Antibacterial Hydrogel for Enhancing Bone Healing

Yuxuan Pang[1†], Lin Guan[2†], Yanlin Zhu[3], Ruijuan Niu[4], Song Zhu[1*] and Quan Lin[2*]

*** Correspondence:** Song Zhu, zhusong1965@163.com

Quan Lin, Linquan@jlu.edu.cn.

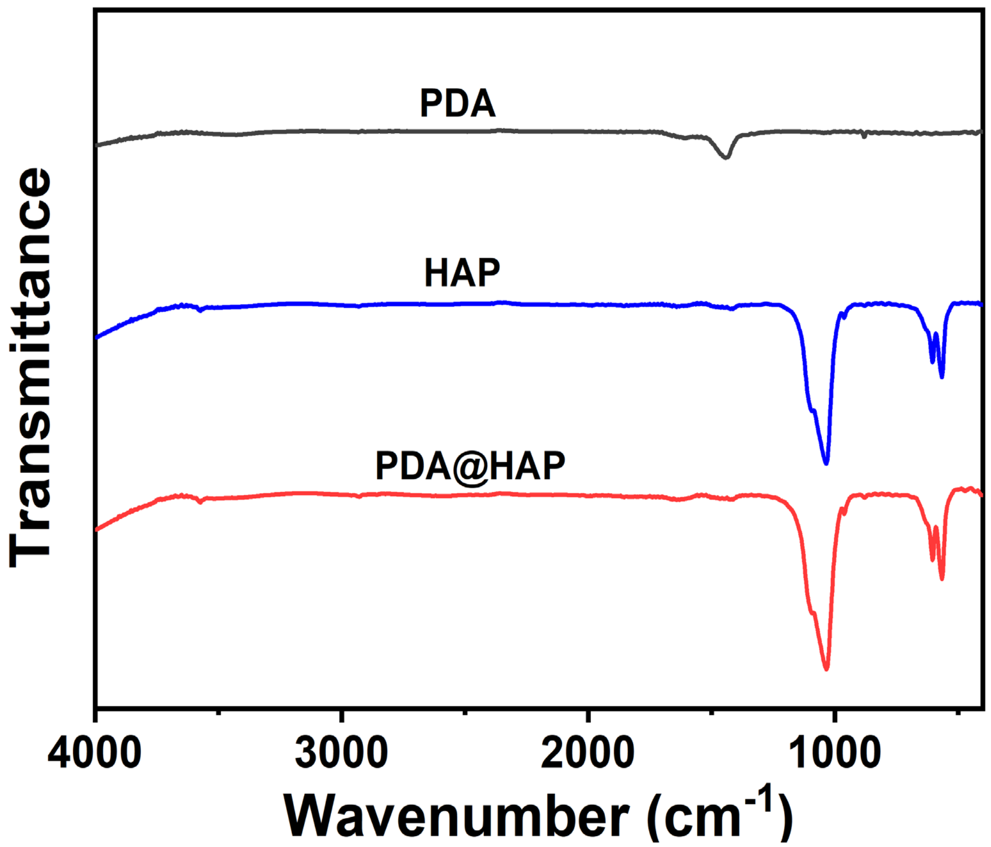

Supplementary Figure 1. The FTIR spectra of PDA, HAP, and PDA@HAP.

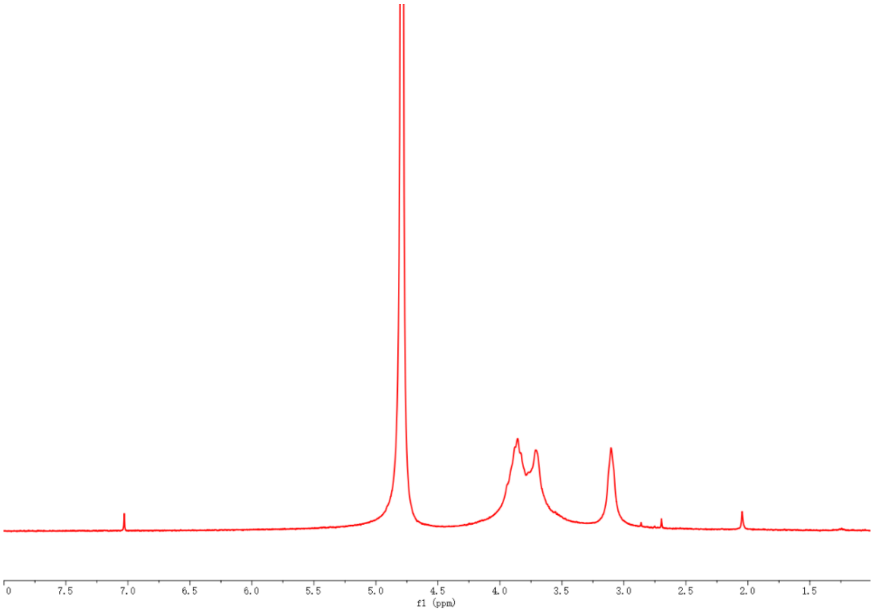

Supplementary Figure 2. The H NMR data of CS-GA.

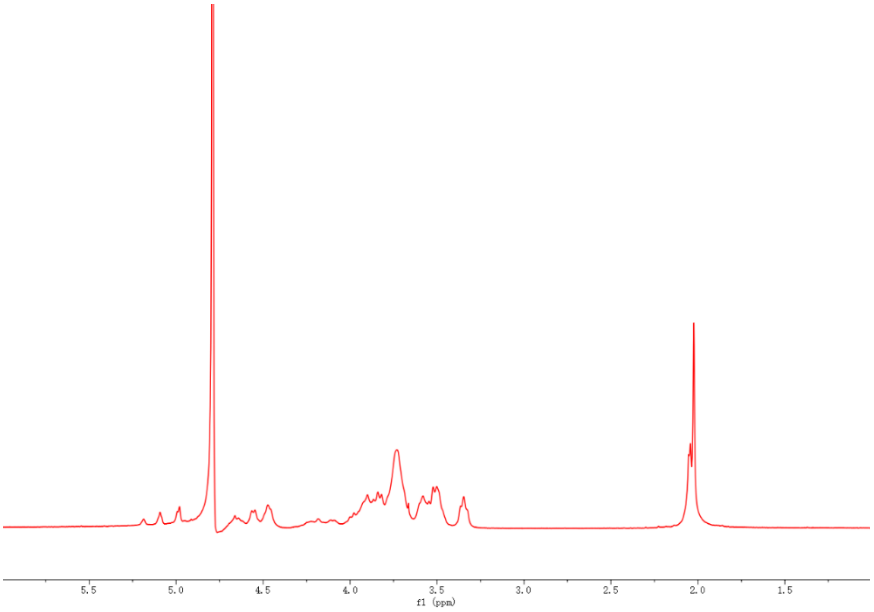

Supplementary Figure 3. The H NMR data of HA-ALD.

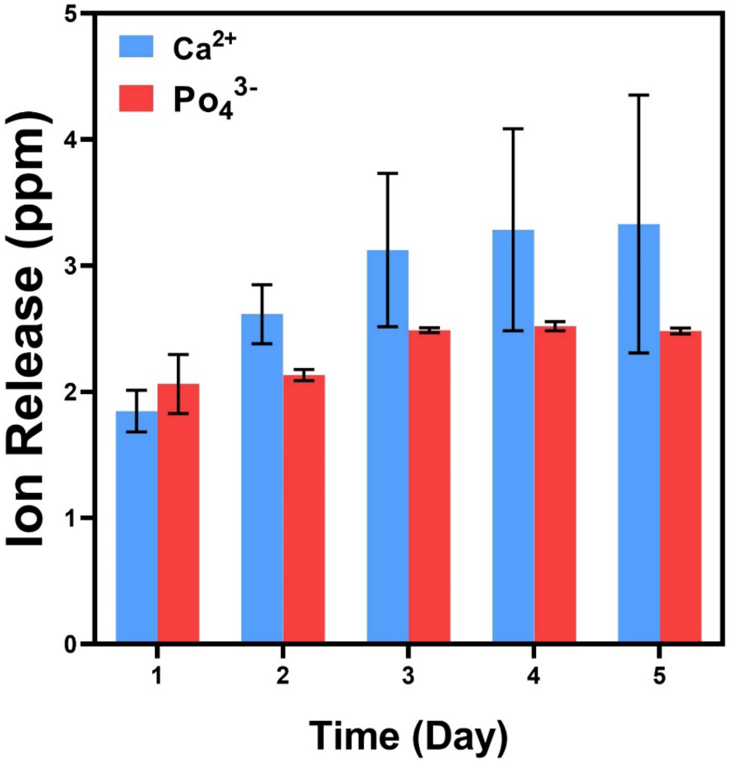

Supplementary Figure 4. *In vitro* release of Ca^{2+} and PO_4^{3-} from the CGH/PDA@HAP hydrogels.

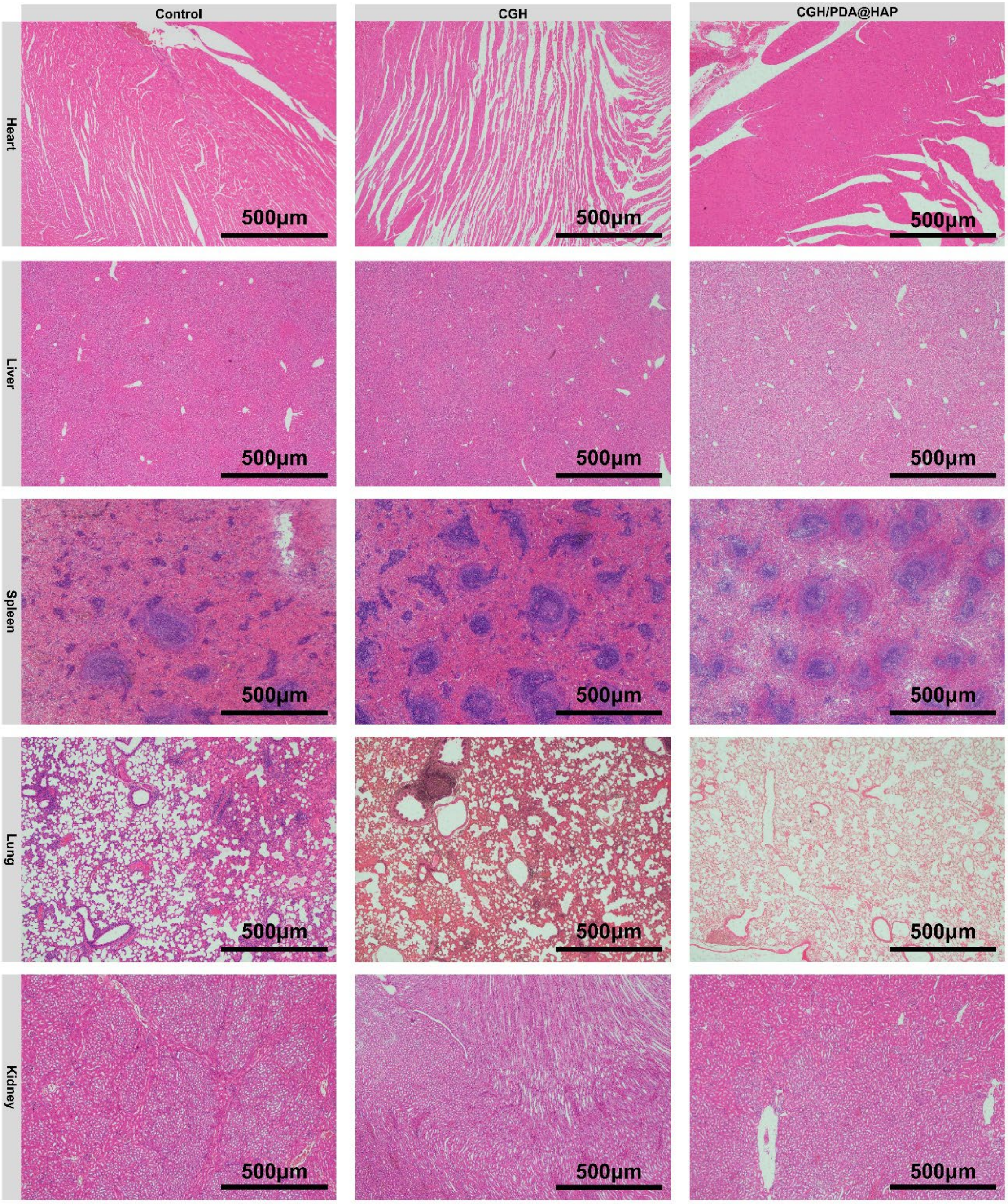

Supplementary Figure 5. H&E staining images of major organs.

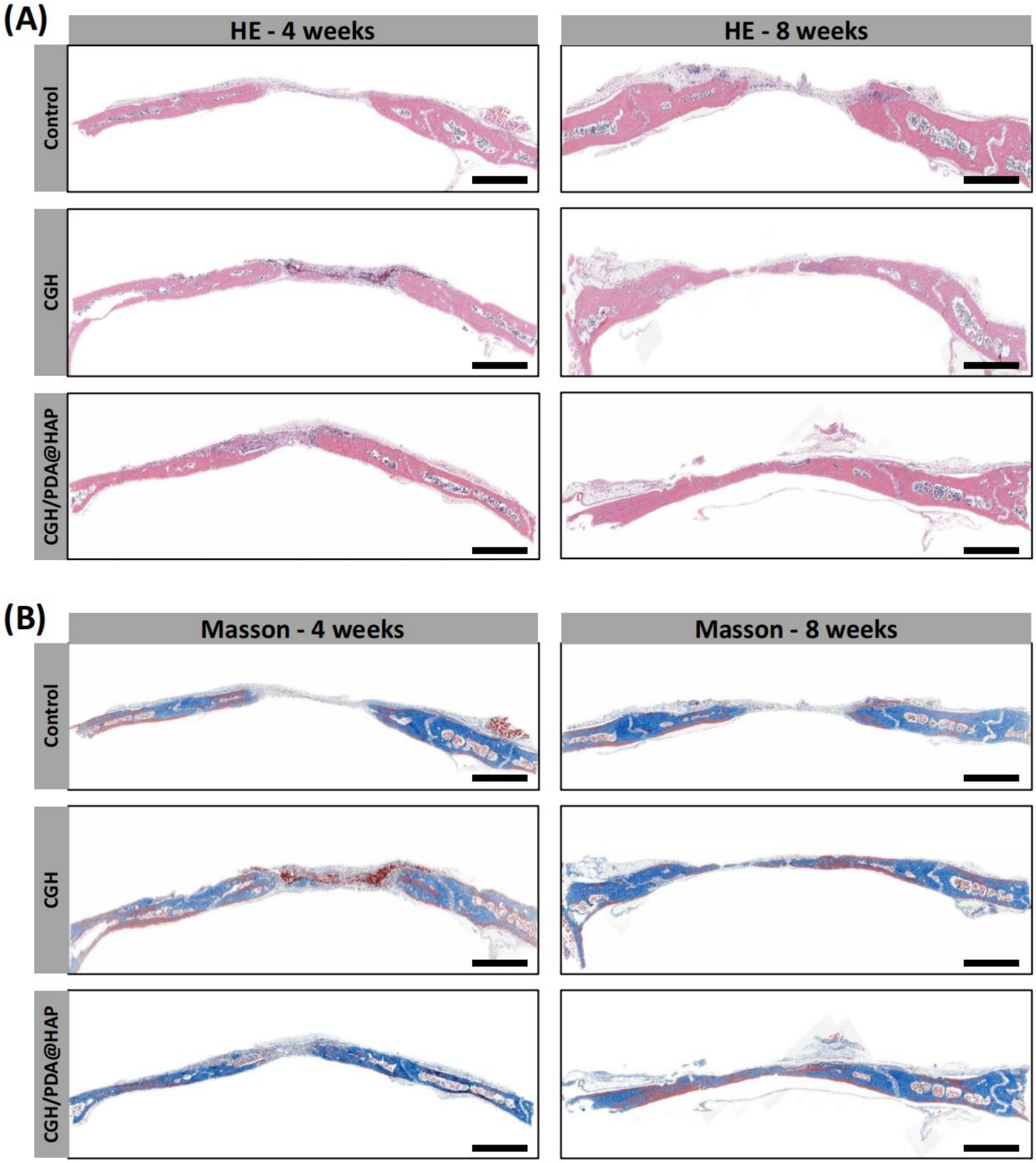

Supplementary Figure 6. The histological images of the full defects. **(A)** HE staining. **(B)** Masson staining. **Scale bar:** 2 mm

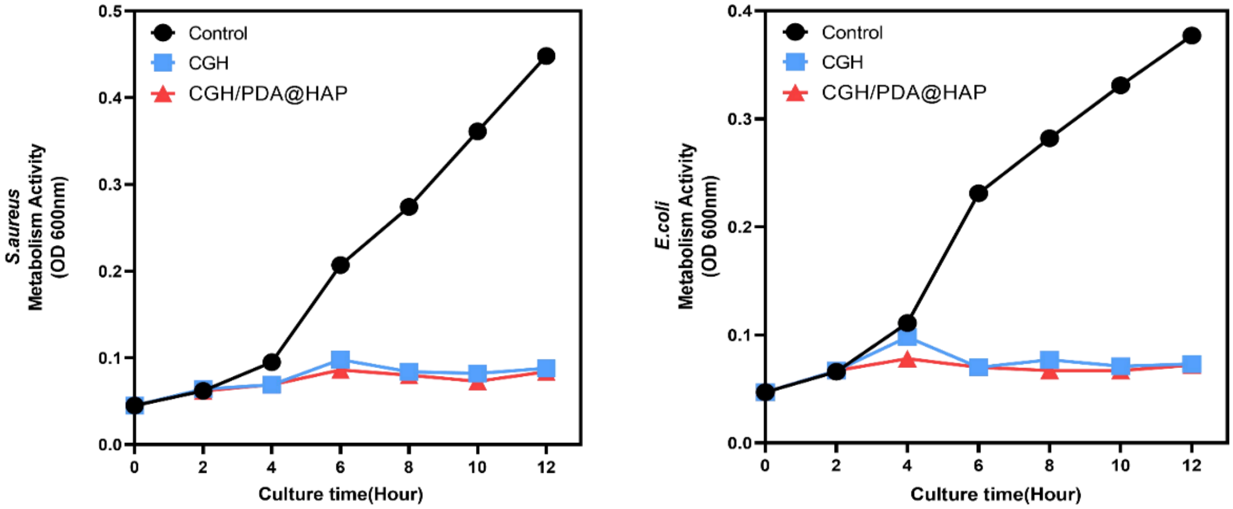

Supplementary Figure 7. Measurement of Time-kill curves to investigate the inhibiting effects of CGH/PDA@HAP and CGH against *S. aureus* and *E. coli* growth.
